# Submolecular Resolution Imaging of P3HT:PCBM Nanostructured Films by Atomic Force Microscopy: Implications for Organic Solar Cells

**DOI:** 10.1021/acsanm.2c01399

**Published:** 2022-06-17

**Authors:** Letizia Liirò-Peluso, James Wrigley, David B. Amabilino, Peter H. Beton

**Affiliations:** † The GSK Carbon Neutral Laboratories for Sustainable Chemistry, School of Chemistry, University of Nottingham, Triumph Road, Nottingham NG7 2TU, U.K.; ‡ School of Physics and Astronomy, University of Nottingham, University Park, Nottingham NG7 2RD, U.K.; § Institut de Ciència de Materials de Barcelona, Consejo Superior de Investigaciones Científicas, Carrer dels Til.lers, Campus Universitari de Bellaterra, 08193 Cerdanyola del Vallès, Spain

**Keywords:** bulk-heterojunction, crystallinity, morphology, organic photovoltaics, scanning probe, XPS

## Abstract

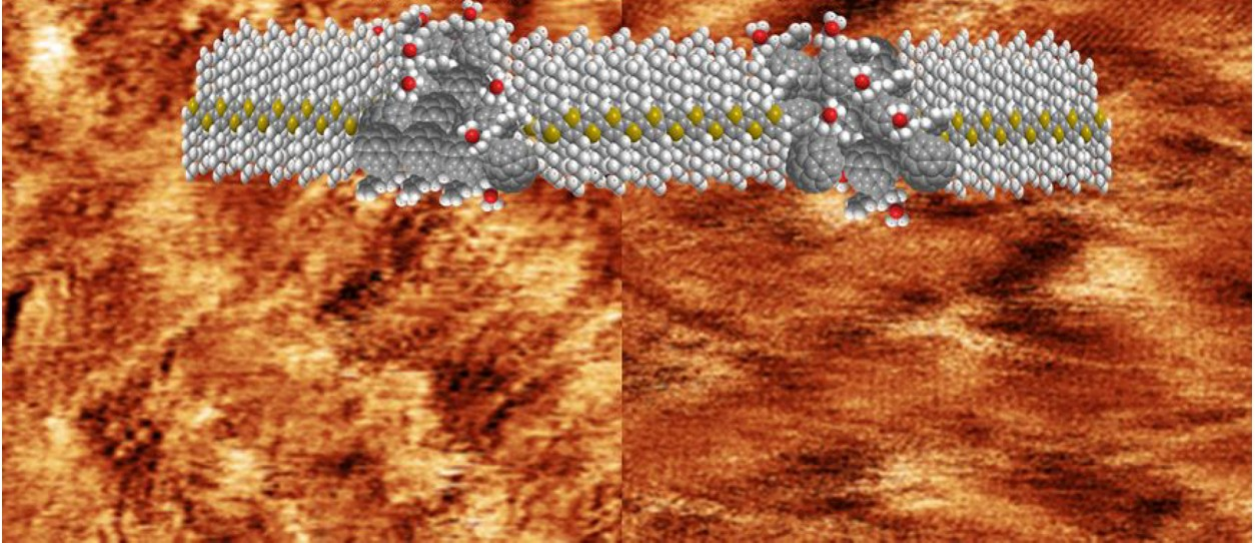

The efficiency of organic bulk-heterojunction (BHJ) solar cells depends greatly on both the bulk and surface structure of the nanostructured bicontinuous interpenetrating network of materials, known as the active layer. The morphology of the top layer of a coated film is often resolved at the scale of a few nanometers, but fine details of the domains and the order within them are more difficult to identify. Here, we report a high-resolution atomic force microscopy (AFM) investigation of various stoichiometries of the well-studied poly(3-hexylthiophene):[6,6]-phenyl C_61_ butyric acid methyl ester (P3HT:PCBM) active layer mixture. Images of the surface were obtained using AC-mode AFM exciting higher-order resonance frequencies of a standard silicon probe, a promising technique for acquiring real-space images of organic-based thin films with nanoscale and even submolecular resolution. We provide firm evidence of the nanoscale organization of the P3HT polymer and of the P3HT:PCBM stoichiometric mixtures at the surface–air interface of the BHJ architecture. Our study shows the characteristic periodicity of the regioregular P3HT identified in the nanoscale domain areas with submolecular resolution. Such areas are then distorted in place when adding different quantities of PCBM forming stoichiometric mixtures. When the samples were exposed to ambient light, the morphologies were very different, and submolecular resolution was not achieved. This approach is shown to provide a precise view of the active layer’s nanostructure and will be useful for studies of other materials as a function of various parameters, with particular attention to the role of the acceptor in tuning morphology for understanding optimum performance in organic photovoltaic devices.

## Introduction

**Scheme 1:**
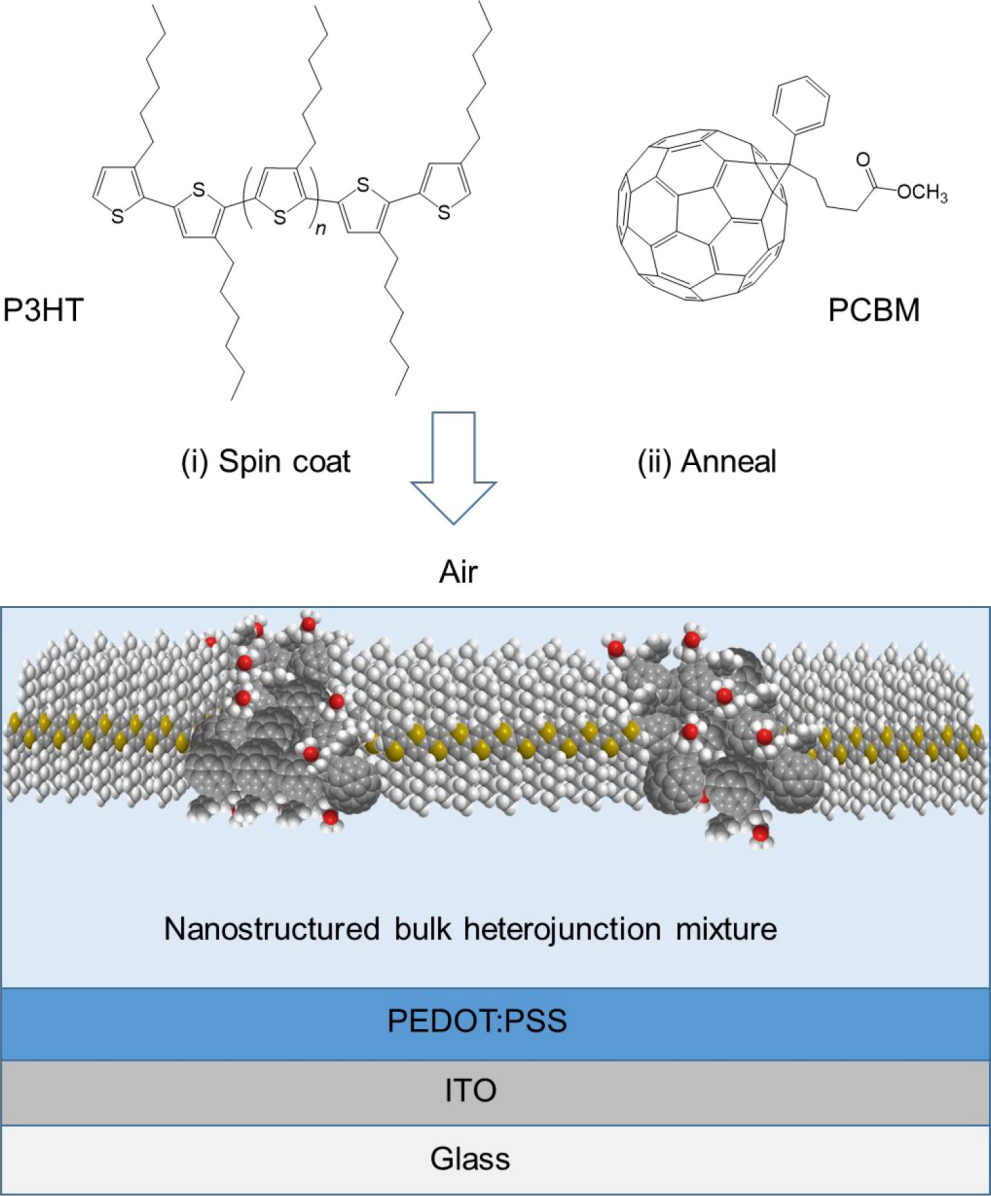
Representation of the Process Used to Prepare the Nanostructured Films of the P3HT:PCBM Composite from a Solution of the Two Components The layers are formed on top of ITO-coated glass previously coated with a layer of PEDOT:PSS, as in many OPV devices.

The development of efficient organic-based photovoltaic devices (OPVs) comprising semiconducting materials in a bulk heterojunction (BHJ) requires high-yielding charge generation and excellent charge extraction toward the electrodes in the systems.^1^ To achieve those requirements, a high contact area between the p-type (electron donor) and the n-type (electron acceptor) semiconductors is necessary; the situation is optimum with nanoscale phase separation that also favors charge separation after short exciton diffusion.^1−3^ Nonetheless, the morphology of the binary mixtures is not easily controlled, and it is sensitive to a wide range of conditions; the nanoscale organization is affected by parameters such as the composition and chemical properties of the molecules, solvent evaporation rate, humidity, and substrate surface free energy.^4−10^ Remarkable advances in OPV performance have been achieved through the introduction of low-band-gap polymers and fullerene derivatives.^11−18^ One of the breakthrough (and most developed) active-material systems comprises a BHJ (Scheme 1) formed by poly(3-hexylthiophene) (P3HT)^19^ and a fullerene derivative (PCBM)^14^ showing a power conversion efficiency (PCE) in the range 3.5–5.0%.^1,3,20,21^ The nanoscale domains and resulting large contact area between donor and acceptor materials lead to effective charge photogeneration and exciton dissociation. Thus, the organization of the two molecules at a nanodomain level influences the generation of polarons, which partially explains the relatively poor PCE in P3HT:PCBM BHJ solar cells when compared with more recent materials (that also have higher visible light absorption leading to greater efficiency).^22^ Consequently, the PCE values of OPVs are strongly dependent on the morphology of the bulk and at the two interfaces of the binary mixture.^1,3,23−25^ Establishing the link between the architecture of the interpenetrating network and the efficiency is difficult but is necessary for the advancement of BHJ photovoltaic systems.^2,26^

The spin-coating deposition of the P3HT:PCBM mixture followed by postdeposition thermal annealing reorganizes the two materials at the nanoscale within the active layer.^6,19^ This process, known as vertical phase separation, arises because of the difference of the surface free energy of the two compounds in contact with the PEDOT:PSS covered substrate, on one side of the active layer, and air, on the other. Also, it promotes the formation of PCBM clusters at the BHJ–air interface of the active layer.^4,5^ The annealing step, with variable time and temperature, favors the thermodynamic achievement of a more energetically favorable state that encourages charge transport through the layers in the device.^6,24,27,28^ However, there has yet to be a submolecular nanoscale investigation of the BHJ surface at its interface with air and how the morphology of that surface changes under certain conditions. The work presented here is a proof-of-concept work focused on the investigation of the surface morphology of spin-coated BHJ mixtures and annealed films, in the case studied with different P3HT:PCBM ratios. We investigated the morphology of the films made of the mixture of the two molecules, spin-coated on a typical OPV heterostructure, and compared their morphology to that of the pure polymer at a nanoscale level.

The acquisition of real-space images at a microstructural level and with a molecular resolution is required to understand the local nanostructure in thin films for OPV applications.^29,30^ Analyses conducted under ambient conditions, by exciting higher-order resonances of an AFM cantilever, provide a valuable route for acquiring real-space images of semiconducting polymers with a submolecular level resolution.^31−33^ In addition, the acquisition of phase channel images, collected from AC-mode AFM, is particularly important for soft materials’ analysis where it can be interpreted as being sensitive to physical properties and compositions that tune the damping of the oscillating cantilever.^2,29,31,32,34−40^ Our study combines a submolecular resolution AFM analysis with a perspective on the prior body of work on P3HT:PCBM mixtures to contribute to the understanding of the intimate connection between nanoscale morphology and functionality and to establish the validity of the scanning probe approach to do so.

## Experimental Section

### Materials

Commercial indium tin oxide (ITO) coated on glass substrates was purchased from Diamond Coatings (0–12 Ohm/cm^2^, 500 nm thickness). The poly(3-hexylthiophene-2,5-diyl) (P3HT, purity of 99.995% and an average molecular weight *M*_n_ = 45,000–65 000 Da) and the phenyl C61 butyric acid methyl ester (PCBM, 99.5% purity) were purchased from Sigma-Aldrich and used without purification. Poly(3,4-ethylenedioxythiophene):poly(styrenesulfonate) (PEDOT:PSS) was purchased from Ossila (1.0–1.2 wt %, aqueous solution).

### P3HT:PCBM Film Preparation

The ITO/glass slides were cleaned by first sonicating with acetone followed by isopropanol and then dried in a N_2_ flux. PEDOT:PSS was filtered through a poly(vinylidenedifluoride) filter (0.45 μm pore size), deposited by spin-coating (40 μL, 3000 rpm for 30 s) onto the ITO–glass substrates, and then dried on a hot plate for 10 min at 130 °C. Solutions of PCBM and P3HT were prepared (1:0, 1:0.8, 1:1.5, 1:2; w/w) with a total concentration of 23 mg/mL dissolved material in chlorobenzene and stirred for 19 h in darkness at 60 °C to promote complete dissolution. The mixtures (60 μL) were spin-coated, by applying 550 rpm for 30 s and an additional 30 s at 3000 rpm for drying onto a PEDOT:PSS film previously prepared on ITO–glass substrates. After the deposition of the P3HT:PCBM mixtures onto the PEDOT:PSS/ITO–glass substrates, the samples were thermally annealed on a hot plate for 15 min at 130 °C. The spin-coating was performed under ambient conditions at room temperature (∼22 °C) using a POLOS200 Advanced-NPP Table-Top spin-coater provided with vacuum chuck.

### AFM Imaging

AFM images were acquired in AC-mode under ambient conditions using an Asylum Cypher S AFM (Oxford Instruments-Asylum Research, Santa Barbara, CA, USA). The images were collected using a Scout 70R cantilever from Nunano (spring constant of 2 N/m and a fundamental resonant frequency in the range 50−70 kHz). To achieve high-resolution images, the cantilever was driven at either the second or third eigenmode, corresponding to resonant frequencies of 300−450 kHz and 900−1200 kHz, respectively. Raw data were processed from .ibw files with Gwyddion, and further processing data were performed using a Python script.^41^

### XPS Analyses

A quantitative elemental characterization of the surface has been provided using an XPS Kratos AXIS Ultra DLD instrument. The chamber pressure during the measurements was 5 × 10 ^–9^ Torr. Wide energy range survey scans were collected at a pass energy of 80 eV in hybrid slot lens mode and at a step size of 0.5 eV, for 20 min. High-resolution data on the C 1s, O 1s, and S 2p photoelectron peaks were collected at a pass energy of 20 eV over energy ranges suitable for each peak and at collection times of 5 min and a step size of 0.1 eV. The X-ray source was a monochromated Al Kα emission, run at 10 mA and 12 kV (120 W). The data were processed with CASAXPS.

## Results and Discussion

### Film Preparation and Atomic Force Microscopy

**Figure 1:**
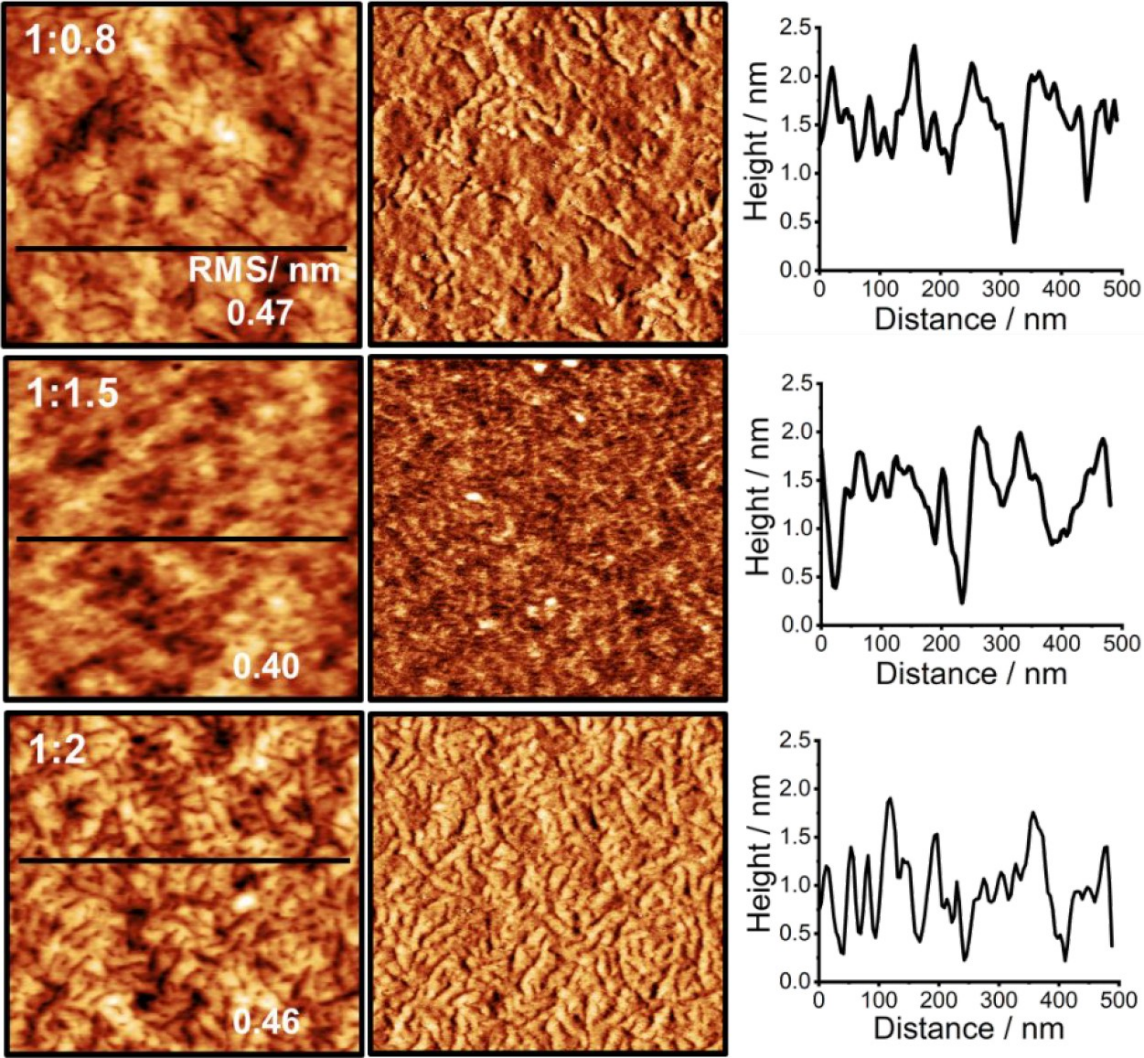
AC-mode AFM images acquired using the first eigenmode of the cantilever (scan size 500 × 500 nm) of P3HT:PCBM blends stored in the dark after preparation. Topography (left column) and phase (right column) images with corresponding black line profiles. Blends: 1:0.8 (top row), 1:1.5 (middle row), and 1:2 (bottom row).

Films of P3HT:PCBM mixtures, the pure P3HT polymer, and PCBM were spin-coated onto thin films (∼50 nm) of the hole alignment layer poly(3,4-ethylenedioxythiophene):poly(styrenesulfonate) (PEDOT:PSS) spin-coated onto ITO–glass substrates, simulating the architecture used for many OPV devices. The PEDOT:PSS substrate provides a reasonably flat interface for the deposition of the active layers (see Supporting Information (SI)). In a characteristic OPV heterostructure, the PEDOT:PSS layer is also included, in part, to align energy levels. Two series of experiments were performed for films prepared with varying ratios of P3HT:PCBM (1:0, 1:0.8, 1:1.5, and 1:2) and analyzed using AC-mode AFM after thermal annealing. One batch of samples was left under ambient laboratory light conditions for at least 24 h, and a second batch was kept in the dark to estimate how films can be influenced by light from a topographical point of view (see Supporting Information for AFM images, Figures S1–S7). Exposure to either light or heat can induce OPV degradation, which can occur through different mechanisms and can induce topographical changes of the sample’s surface.^42^ Micro- (Figures S1–S6) and nanoscale (Figure 1 and Figure S7) topographic and phase images and profiles, extracted from the topography images, of the annealed films using different P3HT:PCBM ratios (cast solution concentration of 23 mg/mL in chlorobenzene) were recorded followed by the analyses of the pure P3HT films.

Focusing on the samples left in the dark (Figures 1 and S1–S5) the AFM images show a fiberlike arrangement where the surfaces appear isotropic and smooth, with a root mean square (RMS) value, calculated over a 1.0 μm^2^ and 500 nm^2^ scan area, on the order of 0.5 nm. Such fiberlike features have been reported previously by tuning other preparation parameters (temperature, annealing time, the drying time of the solvent, and spin-coating) as well as the ratio between the blended donor and acceptor materials.^28,38,39,43^ The chemical and physical properties of the polymer tune its ability to crystallize; therefore, these properties also impact the morphology of the bulk.^44,45^ As the blend ratio is varied, the films display slightly different morphologies with features better resolved in the phase images. In this specific case, dark features in the phase image largely coincide with depressions in the topographic signal. In particular, the AFM images of the 1:2 blend show well-defined nanoscale features that might indicate the presence of PCBM at the air–BHJ interface (see Figure S5, for example). In contrast, the blends exposed to ambient light (see Figures S6 and S7 in the SI) exhibit features reminiscent of globular nanostructures rather than a fiberlike arrangement. Despite the morphological differences, the two batches of samples have similar overall roughnesses.

**Figure 2:**
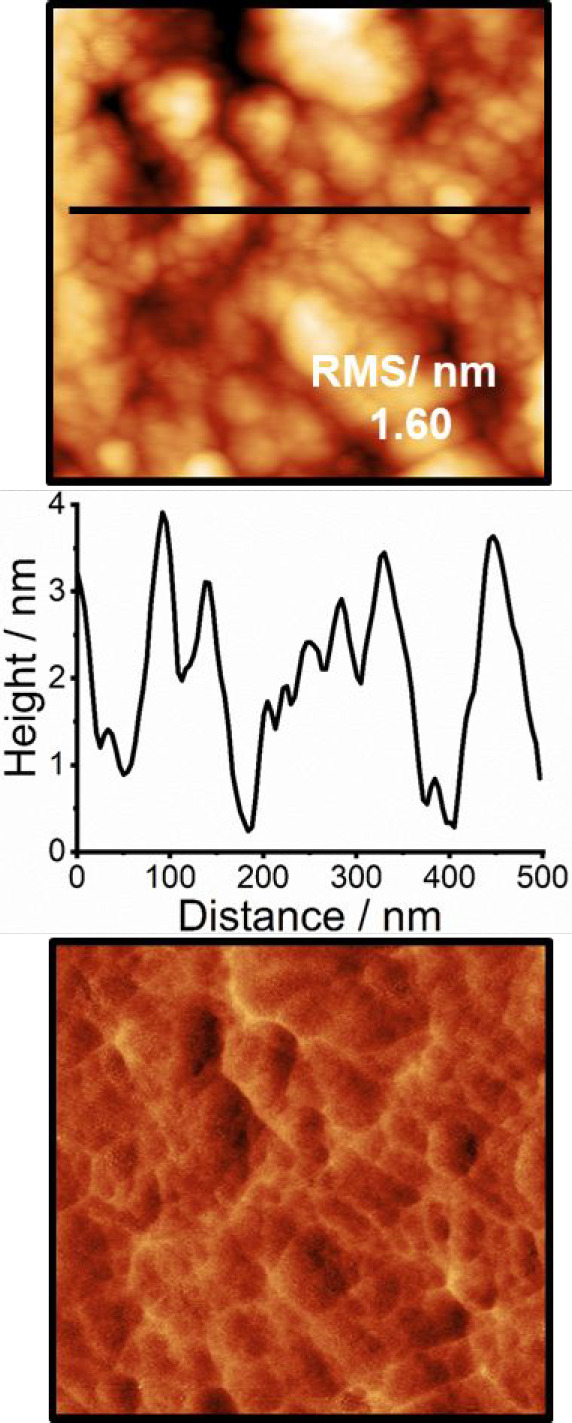
AC-mode AFM images acquired using the first eigenmode of the cantilever (scan size 500 × 500 nm) of a P3HT polymeric film stored in the dark after preparation. Topography (top row) with corresponding black line profile (middle row) and phase (bottom row) images.

**Figure 3:**
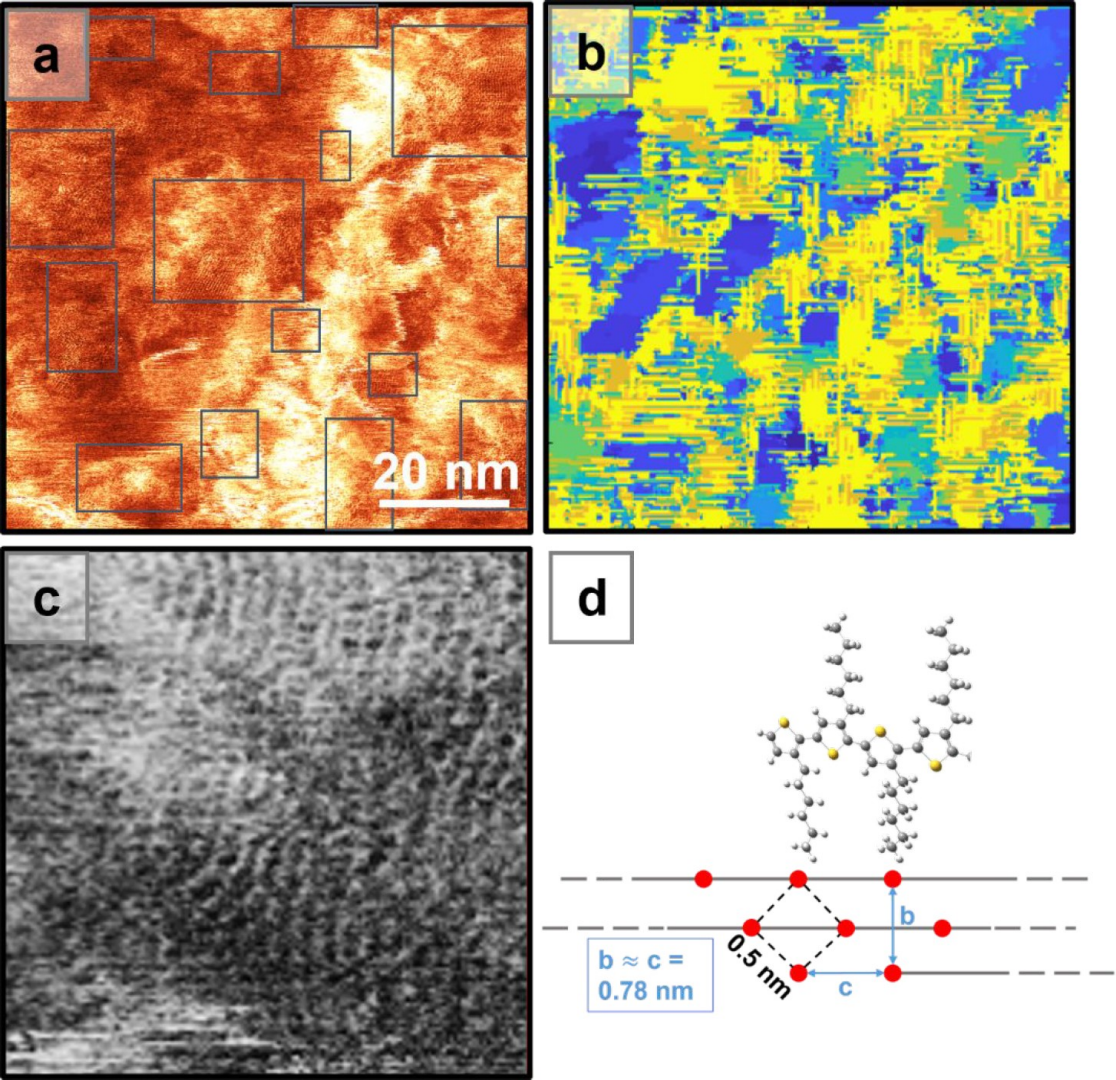
(a) High-resolution AFM phase image of the polymeric P3HT film acquired in AC-mode with a cantilever oscillating at the third eigenmode 927 kHz, scan size (100 × 100) nm. Blue rectangles highlight the regions with periodic order. (b) Color map of the P3HT phase image showing the local periodicities of different areas of the surface, generated using the methodology described in refs (29 and 41). Each pixel is colored depending on the periodicity of the strongest FFT peak of the kernels: the blue regions have periodicity corresponding to P3HT; other colors are regions of deviation or where noise dominates the periodic signal. (c) Zoom of one of the highlighted regions in part a showing the polymeric P3HT chains. Gray scale is used for highlighting the arrangement of the chains. (d) Schematic drawing of the P3HT arrangement perpendicular to the surface of the film and the periodicity of the square lattice resolved.

In addition, films with only the P3HT polymer or PCBM were prepared. The PCBM films (Figure S8) deposited under the same conditions show a dense mesh of crystallites with well-defined edges. The P3HT films were analyzed at the microscale level (Figure 2) followed by the acquisition of high-resolution images (Figure 3).

The pure P3HT polymer film (Figure 2) has a roughness four times larger (∼1.6 nm) than the films prepared by blending with the fullerene derivative. The submolecular AFM analysis (Figure 3), performed on different areas of the film (see Figure S9 in the SI for more images), shows amorphous regions interspersed with ordered domains (highlighted by rectangles) in which the polymer forms islands of the order of 20 nm in diameter (Figure 3a). Within these islands the apparent square order (Figure 3c) is associated with the termination points of the alkyl side chains attached to the poly(thiophene) backbone and is consistent with polymeric chains running parallel to each other as discussed below.

The P3HT component, in common with other macromolecules, has many structural configurations resulting from the conformational freedom around the bond linking the thiophene units, leading to inhomogeneous organization in the bulk material. The system is often microcrystalline, containing regions of regular packing interspersed with less ordered areas.^46−48^ Korolkov et al.^29^ resolved ordered regions within domains of a P3HT spin-coated film on a PEDOT:PSS film on a mica substrate. The order was identified as an approximately square lattice with a lattice constant of 0.55 nm with a variation of the orientation of the lattice across the surface. Figure 3d shows a schematic indicating the origin of this periodicity. P3HT has been shown to form lamellar domains in which the plane of the poly(thiophene) backbone is perpendicular to the free surface. The separation of neighboring planar units is 0.39 nm.^49^ The anchoring points of the alkyl chains on a common edge of the poly(thiophene) backbone are separated by 0.78 nm, corresponding to the repeat length of the polymer. As shown by Kayunkid et al.,^49^ adjacent poly(thiophene) strands are relatively displaced by 0.55 nm so that the anchoring points of the alkyl chains form an array represented by the red dots in Figure 3d. The terminal methyl groups of the alkyl chains form an array at the surface which is revealed in our AFM images.

The samples prepared in this work show a similar organization in the regions where it is possible to resolve these features (Figure 3), which were quantitatively evaluated by performing a map of the local periodicity of the surface (Figure 3b) with a similar method to that reported by Summerfield et al.^41^ The deep blue areas, in Figure 3b, represent periodicities across the blue squared highlighted regions corresponding to the expected lattice period of P3HT with a real-space value in the range 0.45–0.56 nm. These findings are also supported by previous diffraction and spectroscopic studies where the films made of regioregular P3HT form semicrystalline domains. The plane of the backbones is perpendicular to the surface forming a π-stacked in-plane organization with lattice dimensions approximated to a square containing two P3HT chains per unit cell (at the vertices and the center).^49^ In this arrangement, neighboring π-stacked P3HT chains are arranged with an “up” sulfur atom aligned to a “down” sulfur atom in adjacent polymeric chains (see schematic in Figure 3d).

**Figure 4:**
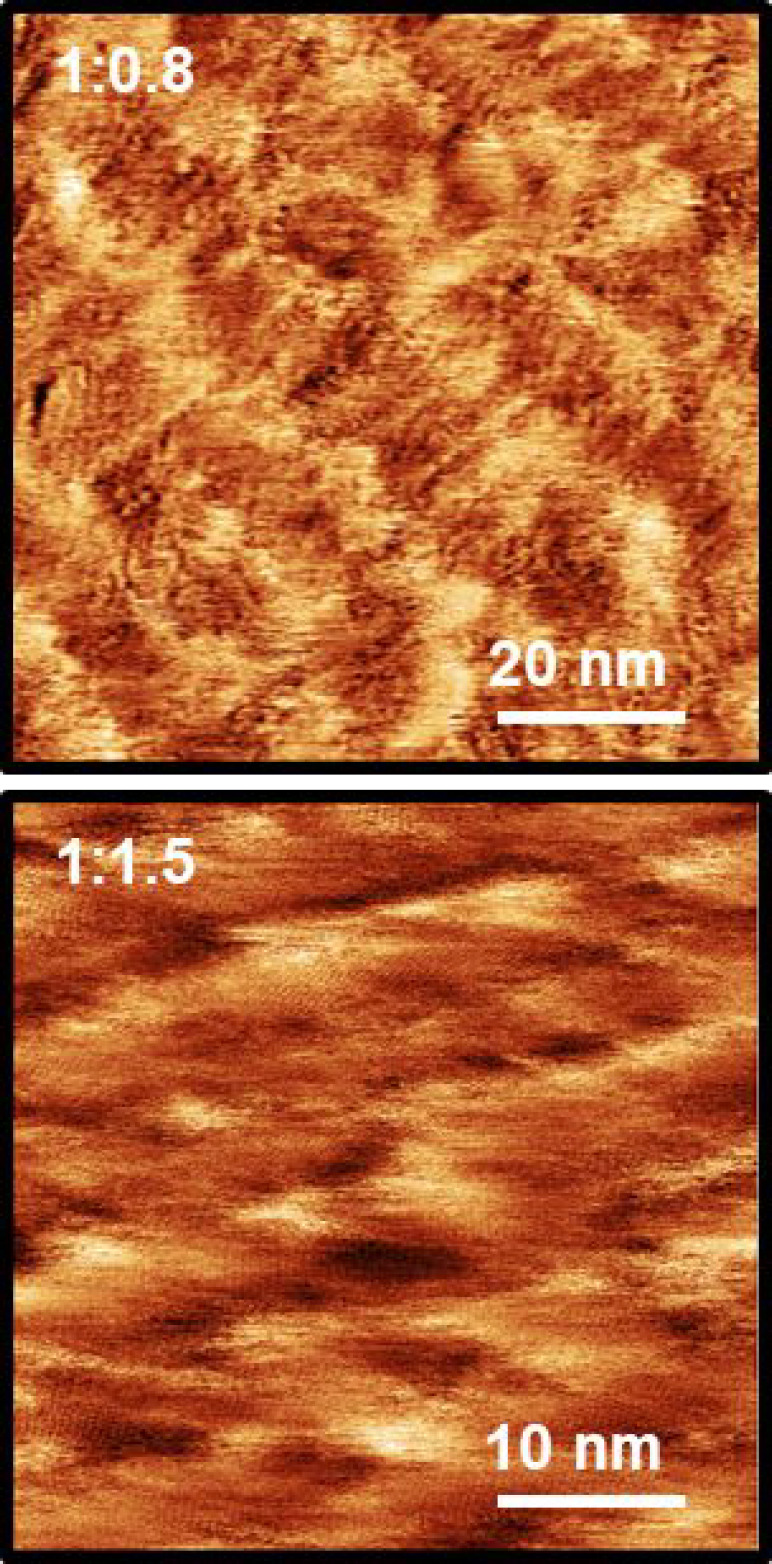
AC-mode AFM phase images of the P3HT:PCBM blends, left in dark conditions, 1:0.8 (top) and 1:1.5 (bottom), acquired using high-frequency modes of the cantilever. Probe oscillating at the second eigenmode (406 kHz): 1:0.8 ratio, scan size (100 × 100) nm; 1:1.5 ratio, scan size (50 × 50) nm.

A similar analysis of the surface of the blends of P3HT:PCBM at 1:0.8 and 1:1.5 ratios (Figure 4), left in either dark or ambient laboratory light conditions, was then recorded in an effort to understand the influence of the fullerene acceptor on the P3HT surface arrangement. The blends left in dark conditions revealed nanosized domains similar to the polymer packing in Figure 3 with a local periodicity in the range 0.42–0.44 nm when calculated using the processing algorithm (see Experimental Section) and interspersed with regions with no obvious order. On the contrary, the samples stored under ambient light in the laboratory did not show any kind of regular arrangement over the surfaces investigated (see Figures S6 and S7 in the SI for the AFM analyses), an effect believed to be caused by photoinduced oxidation of the blend.^50^ For the samples stored in the dark, the significant decrease of the square lattice constant might be attributed to a distortion in the regularity of the domains with respect to the pristine P3HT film. There is no obvious difference in the coverage of ordered domains between the pure polymer film and the materials incorporating the fullerene, although the domain sizes are extremely variable (see Figures S10–S13 in the SI for the AFM analyses).

**Figure 5:**
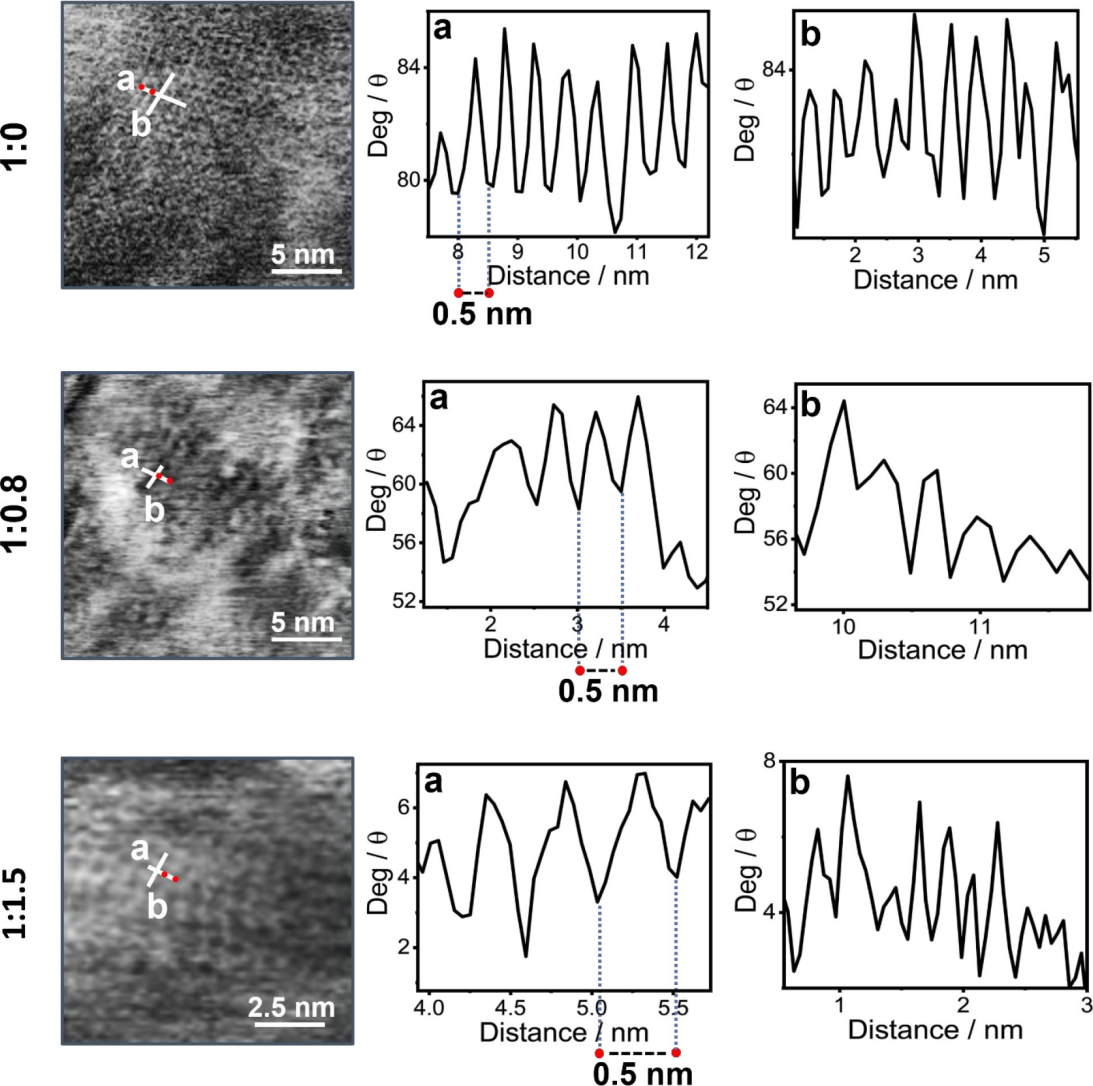
Detail of the nanometric domains showing the arrangement of the polymer and its lattice constant with corresponding line profiles a and b to the right of the AFM images, where perpendicular white lines in the micrographs show the positions of the analyses in the graphs. Gray scale is used here to highlight the visible features.

A comparison of the ordered domains in the pure polymer and the blends of P3HT:PCBM at the 1:0.8 and 1:1.5 ratios (Figure 5) shows areas where the spacing along the backbone is identical, as it should be for the regioregular macromolecule. The presence of the square lattice is seen because the perpendicular profiles have very similar periodicities, although there is an evident distortion along one direction at the edge of one domain for the 1:1.5 ratio sample (Figure 5). This observation indicates that the acceptor is loaded into the polymer at some level, a feature seen by others using other techniques.^51,52^

### X-ray Photoelectron Spectroscopy

**Figure 6:**
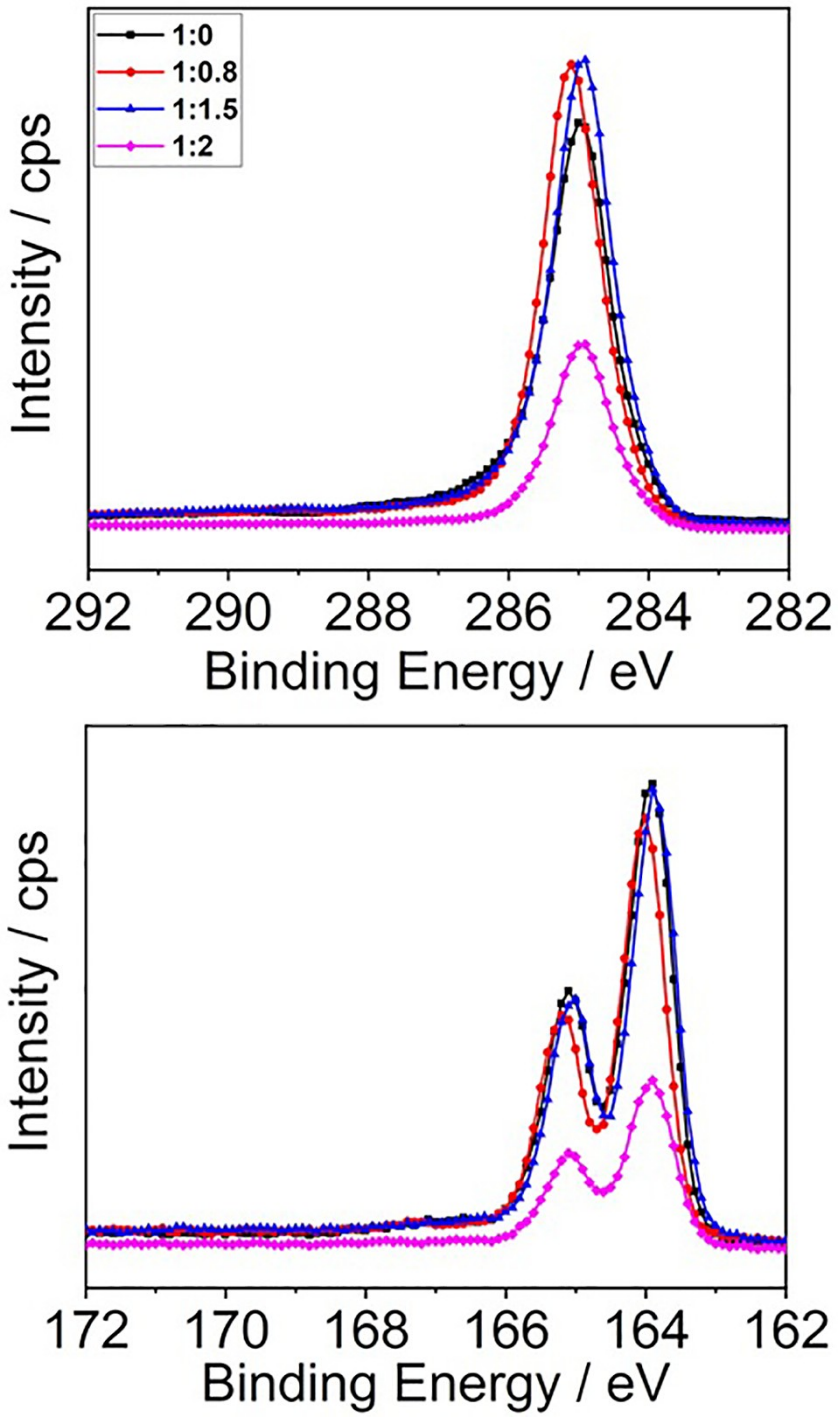
XPS spectra (counts per second, cps vs binding energy, B.E.) for 1:0, 1:0.8, 1:1.5, and 1:2 P3HT:PCBM. C 1s (top row) and S 2p (bottom row) spectra.

We clarified the compositional details of the BHJ–air interface of the stoichiometric films by performing X-ray photoelectron spectroscopy (XPS, see Figures S14–S16 in the SI for details). The variation of a few electronvolts in the positions of both the C 1s (shift of 0.9 eV related to the alkyl chain and 0.7 eV to the methoxy C) and O 1s (0.4 eV related to the carbonyl group and 0.3 eV to the methoxy) peaks, compared to their positions in the film made only of the PCBM fullerene derivative (C 1s, 285.6 eV alkyl chain, 287.1 eV methoxy C; O 1s, 532.4 eV carbonyl group, 533.7 eV methoxy group; see Figure S14 and Table S1 of the SI), confirms the presence of the electron donor P3HT at the film–air interface, interacting with the fullerene derivative. Looking at the S 2p region of the different blends as the percentage of PCBM increases in the mixture, there is a corresponding decrease of the signal intensity for both components at 164.2 and 165.4 eV associated with the thiophene ring (Figure 6). A quantitative analysis of the carbon, oxygen, and sulfur contents on the surface of each film by XPS confirms the presence of both components at the atmosphere–blend interface (Figure S16 and Tables S2 and S3).

## Discussion

**Figure 7:**
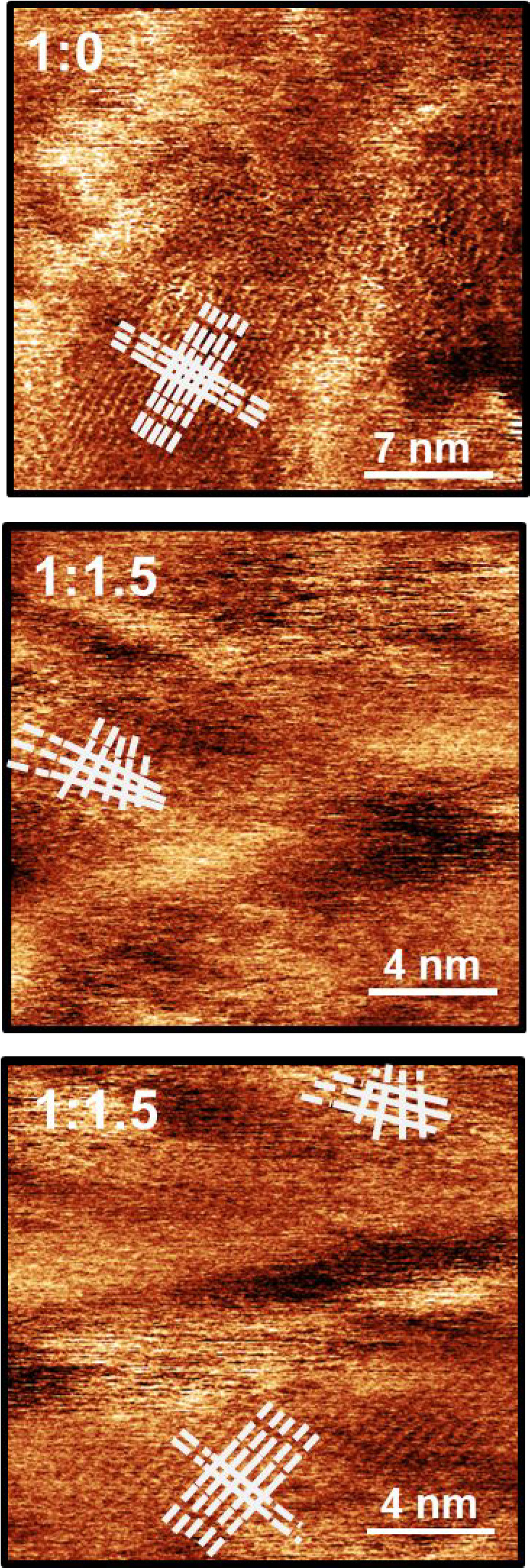
High-resolution phase images of the films of pristine P3HT and the 1:1.5/P3HT:PCBM mixture showing domains with the poly(thiophene) chains running parallel to each other drawing its square lattice and areas where these chains diverge in the 1:1.5/P3HT:PCBM ratio highlighted with white lines.

Our AFM analysis combined with knowledge from previous studies enables us to show that the two components organize at the film–air interface of a characteristic OPV heterostructure in regions of high degree of disorder interspersed with ordered nanometric domains. First, we identified the local periodicity within the domains for spin-coated films made of the neat P3HT polymer, ascribable to the dimensions of the P3HT unit cell previously reported.^29,49^ The addition of different percentages of the PCBM molecule to give the mixtures impacts the organization of the two molecules at the active layer surface–air interface. Thus, the PCBM might be responsible for the distortion in the orientation of the P3HT spacing in the nanometric crystalline domains with respect to the normal to the surface with an apparent change in the square lattice value. That distortion is evident in the AFM image shown in Figure 7, where although some of the larger domains are regular, other smaller domains show irregular spacing of the polymer chains. That distortion may be a result of the small domain size, a consequence of the high proportion of PCBM in the mixture,^53^ where the polymer chains have not crystallized into their most favorable structure at the surface after annealing.

Taking all the variables into account, a BHJ system has a complex 3D morphology within the active layer,^2,39,54−57^ and the postdeposition annealing treatment represents a key step for reaching a thermodynamically more stable state.^23−25,58^ The phase separation process of the two materials codeposited from solution, promoted by the postdeposition thermal annealing treatment,^23,25,54^ results in the formation of nanometric P3HT domains at the air–BHJ interface and in the redistribution of the PCBM within the bulk.^59−62^ This process has been explained using the pseudo-dielectric function of the films as well as ellipsometry and absorption spectroscopy techniques, performed on the blend films, where a specific electronic transition is responsible for the increased P3HT interchain interactions on the surface, which leads to the crystallization of the polymer.^6,27^ In addition, kinetic and cross-sectional studies have shown that the thermal treatment promotes the demixing between the P3HT and the PCBM as a function of their surface free energies and a change in volume percentage distribution of the crystalline P3HT along the 3D architecture of the film.^2,39,54−57^ Such structural reorganization affects also the movement of the PCBM through the bulk. Nicolet and co-workers demonstrated that regions with lack of order can act as nucleation sites where the diffusion of the PCBM molecules through the matrix, to reach the film–air interface and aggregate, becomes possible, forming nanoscopic PCBM agglomerates.^6,44,45,54,57,59,63^ This last consideration is supported by the characteristic globular features seen by AFM (Figure S7 in the SI) in the samples stored under ambient light after annealing. The optical micrographs of these films (see Figures S17–S19 in the SI) show the presence of elongated shape crystals surrounded by a lighter colored depletion zone,^28,44,54,64,65^ and their growth can be modulated accordingly to the conditions used.^54,66−68^ Particularly, the use of chlorobenzene as solvent favors a needle-like shape; increasing the content of fullerene and annealing the system allows the growth of structures hundreds of microns in size,^54,66,67^ as reported for the blend 1:2 P3HT:PCBM, which shows the largest crystals of the series and the absence of ordered domains from our AFM analyses.

## Conclusion

Our study has shown the utility of AC-mode AFM, using higher-order resonance frequencies of the oscillating cantilever, to help create a unified picture of organic composite films by revealing with high precision the topographical organization at their interface with air, in this case for the P3HT:PCBM active BHJ layer architecture that has been so important for the development of organic solar cells. For the first time, we reported the nanoscale organization of mixtures with different ratios (1:0.8 and 1:1.5 P3HT:PCBM), and we were able to compare these findings with the organization of the pristine polymer on a BHJ surface structure. The analyses performed on the images acquired have confirmed that the polymer forms nanometric islands where it is ordered and highly oriented with a square lattice value of 0.5 nm. The addition of different percentages of PCBM apparently distorts the polymer organization. This effect is possibly a result of an influence of the acceptor—that is clearly present at the atmosphere–blend interface—on the domains of the polymer compared to the film of pristine P3HT.

The real-space organization of the surface of the active layer as a mixture between P3HT and PCBM reveals the distribution of both materials over the surface at the interface with air, which is also confirmed by XPS analysis and optical micrographs. This accounts, in part, for the poor PCE of this important system, though the absorption characteristics of the materials and the nanostructure in the bulk of the film are also vitally important. In fact, the ideal configuration would be dictated by the P3HT being in contact with the PEDOT:PSS interface rather than at the atmospheric interface as we found. This information indicates that for this particular annealed blend the best configuration, in terms of OPV performance with appropriate choice of electrode materials and energy alignment layers (as proven in devices^69,70^), would be the inverted configuration. We expect that similar analyses on other material blends will afford vital information concerning device design and material nanostructuring as a function of material composition. We also confirmed that light has a dramatic effect on the morphology of the film, where AFM images of the sample after light exposure do not show the presence of ordered polymer domains, which is surely an important effect concerning the response of the devices under operational conditions.

## Supporting Information Available

VASE measurements on a PEDOT:PSS layer deposited on substrates. Micro- and nanoscale AFM images of films made of P3HT:PCBM mixtures and of the P3HT. XPS data analysis of films of pure PCBM and of P3HT:PCBM mixtures. Optical micrographs of films made of P3HT:PCBM mixtures. (PDF)

## Dedication

This paper is dedicated to Sir J. Fraser Stoddart on the occasion of his 80th birthday.

## Abbreviations

AFM: atomic force microscopy
BHJ: bulk heterojunction
P3HT: poly(3-hexylthiophene)
PCBM: [6,6]-phenyl C61 butyric acid methyl ester
OPV: organic-based photovoltaics
PCE: power conversion efficiency
PEDOT:PSS: poly(3,4-ethylenedioxythiophene):poly(styrenesulfonate)
VASE: variable angle spectroscopic ellipsometry
RMS: root main square
XPS: X-ray photoelectron spectroscopy
