# Supplementary material for: Submolecular Resolution Imaging of P3HT:PCBM Nanostructured Films by Atomic Force Microscopy: Implications for Organic Solar Cells
Source: ACS Appl Nano Mater. 2022 Jun 17;5(10):13794–804. doi: 10.1021/acsanm.2c01399 (PMC9623582; doi:10.1021/acsanm.2c01399)
Supplement: Supplementary file 1 — an2c01399_si_001.pdf [file an2c01399_si_001.pdf]

Supporting Information

Sub-Molecular Resolution Imaging of P3HT:PCBM Nanostructured Films by Atomic Force Microscopy: Implications for Organic Solar Cells

Letizia Liirò-Peluso,[a,b] *James Wrigley,*[b] *David B. Amabilino,*[a,c*] *Peter H. Beton*[b*]

* Corresponding authors. E-mails: amabilino@icmab.es and peter.beton@nottingham.ac.uk

[a] The GSK Carbon Neutral Laboratories for Sustainable Chemistry, School of Chemistry, University of Nottingham, Triumph Road, Nottingham NG7 2TU, UK

[b] School of Physics and Astronomy, University of Nottingham, University Park, Nottingham NG7 2RD, UK.

[c] Institut de Ciència de Materials de Barcelona, Consejo Superior de Investigaciones Científicas, Carrer dels Til.lers, Campus Universitari de Bellaterra,08193 Cerdanyola del Vallès, Spain.

Variable angle spectroscopic ellipsometry (VASE) measurements for the PEDOT: PSS layer deposited on ITO/glass (BK7) substrate

The variable angle ellipsometric response of both the ITO/glass and the PEDOT: PSS layer were satisfactorily fitted using dielectric functions provided in the JA Woollam CompleteEase (v6.62) materials library. The commercially available ITO layer on the glass substrate (Diamond Coating BK7) has a confirmed thickness of 500 nm. The PEDOT: PSS layer, deposited by spin coating on the substrate, has been modelled using anisotropic (uniaxial) dielectric functions at photon energies between 0.7eV and 4 eV. The sample was measured using focus probes with an elliptical spot that had a minor diameter of 300 microns at 55°, 60° and 65° angles of incidence.

First, the ITO-glass substrate was modelled, fitting the data over the full range of the measurement (0.734 eV – 6.473 eV) using an optical model, ITO (GenOsc) in the JA Woollam CompleteEASE (v6.62) library to describe the ITO response and the library function for BK7 glass. The ITO model comprises a Tauc-Lorentz model with the additional terms of a Gaussian oscillator at high energy and a Drude oscillator to describe the response at photon energies below 1.5eV. This model was then used to describe the substrate onto which the PEDOT:PSS film was spin coated. The film itself was modelled as having uniaxial anisotropy. The models were based on the ordinary and extraordinary dielectric functions provided for PEDOT in the JA Woollam CompleteEase (v6.62) library. To estimate the uniqueness of the model, the thickness of the PEDOT: PSS layer was systematically varied from the best fit position and the other fitting parameters refitted to the data. The results suggest that the mean-square-error was altered by 10% for a thickness variation of $\pm 5\,nm$. Therefore, the thickness of the PEDOT: PSS layer (an average over the 300 micron diameter spot size) was estimated to be 43 $nm \pm\ 5\,nm$.

AFM micro- and nano-scale images of the mixtures left either in dark and ambient light conditions.

Stored in the dark

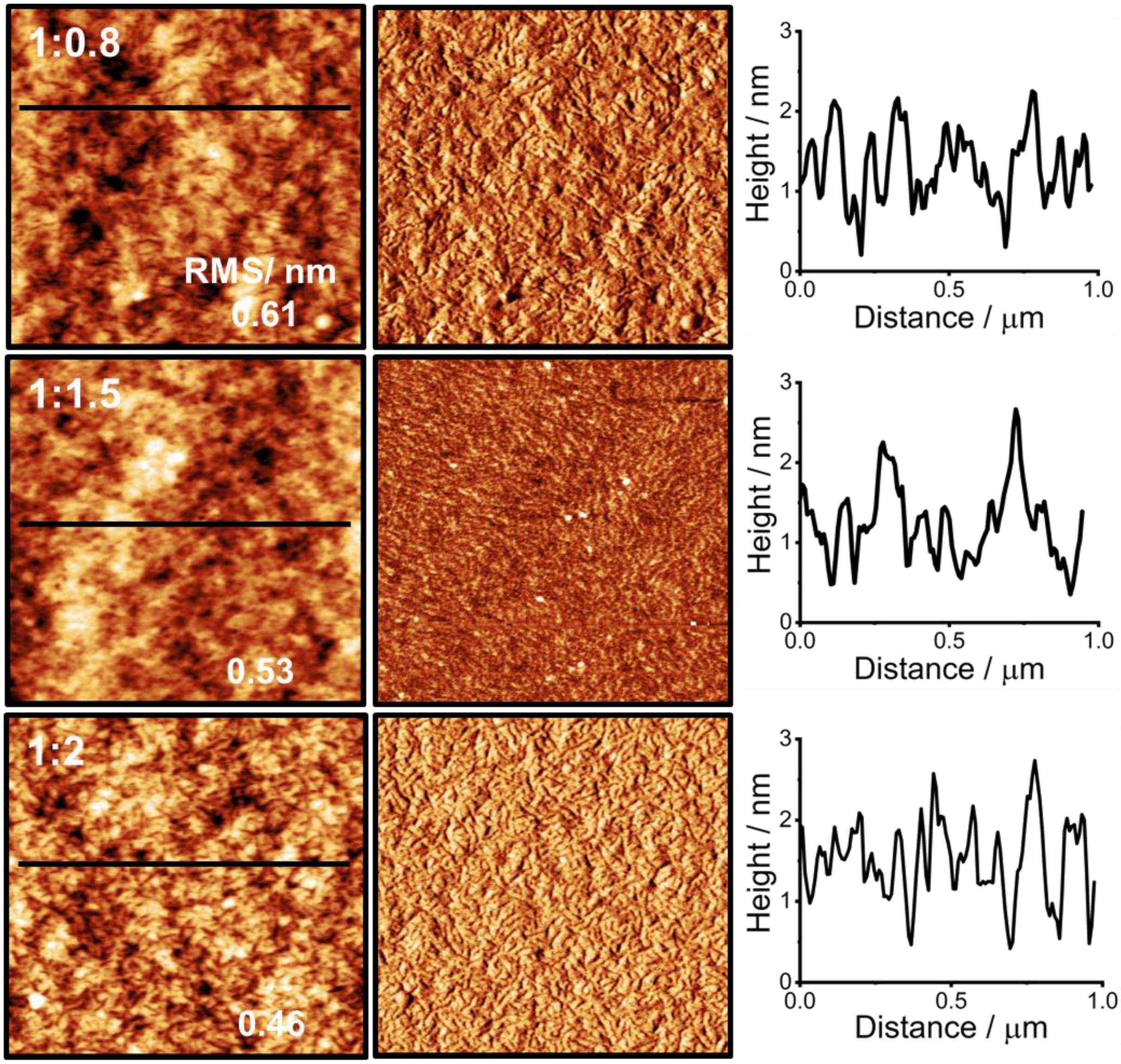

Figure S1. AC-mode AFM images (scan size 1 x 1 µm) of P3HT:PCBM blends stored in the dark after preparation. Topography (left column) and phase (right column) images with corresponding black line profiles. Blends: 1:08 (top row), 1:1.5 (middle row), 1:2 (bottom row). Images were acquired using the first eigenmode of the cantilever.

Stored in the dark

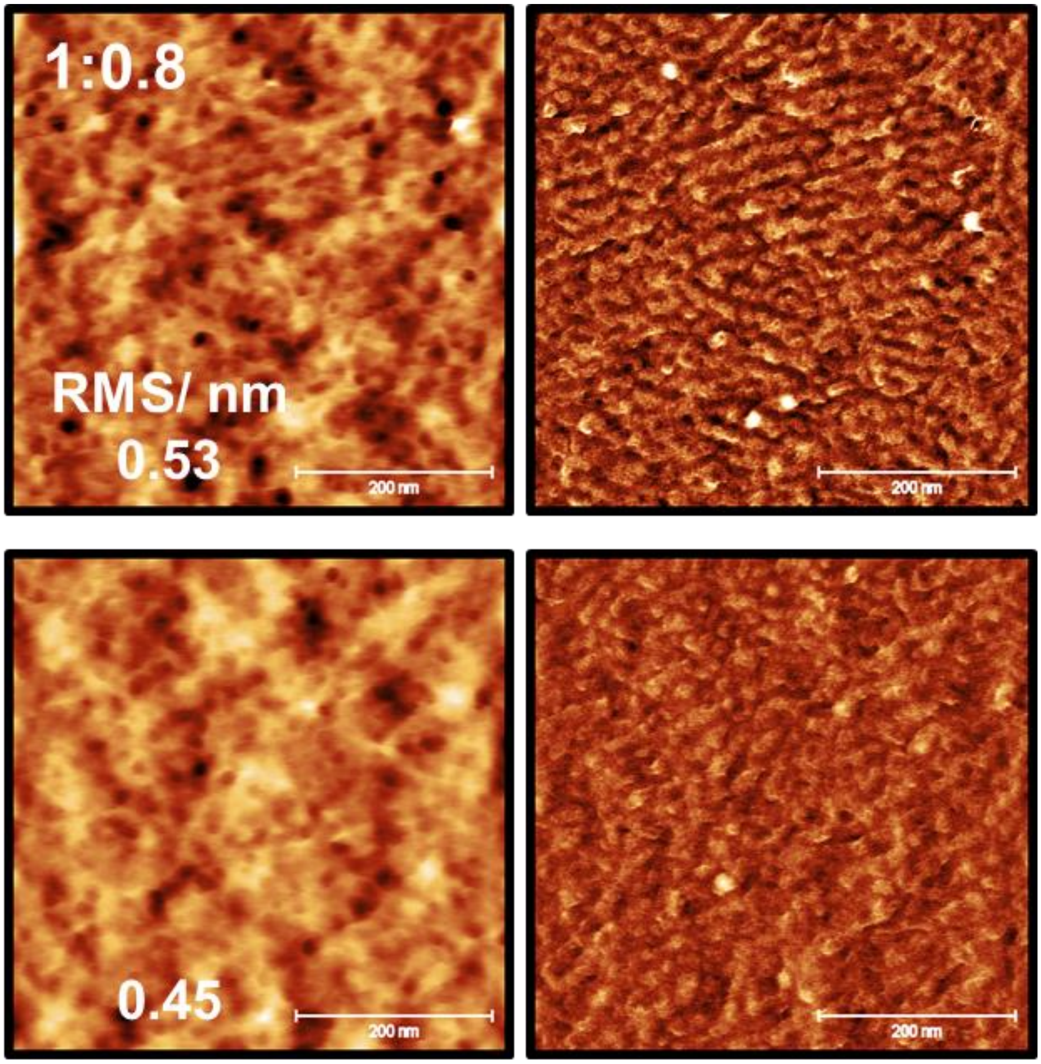

Figure S2. AC-mode AFM images (scan size 500 x 500 nm) of the 1:0.8 P3HT: PCBM blend stored in the dark after preparation. Topography (left column) and phase (right column).

Stored in the dark

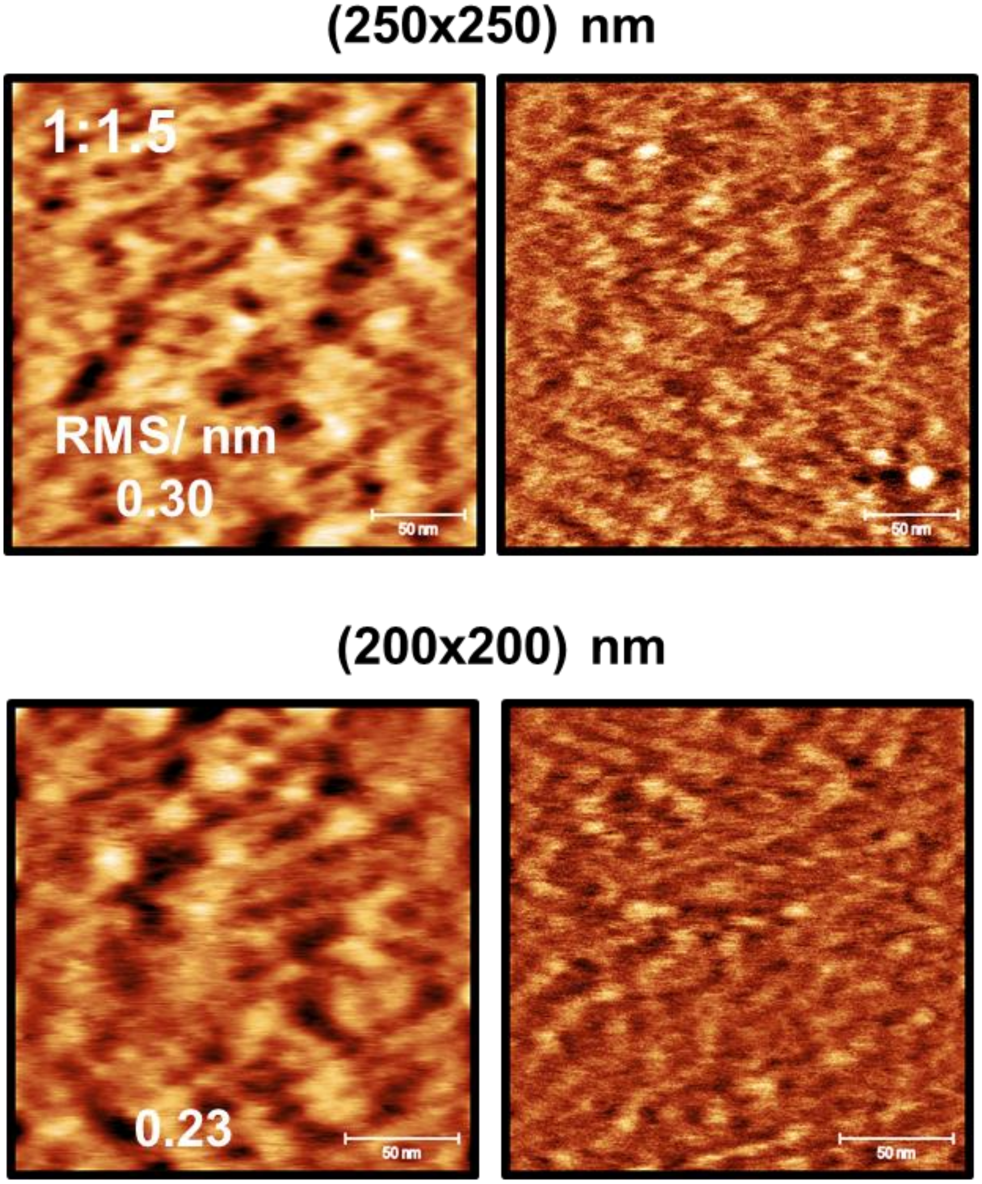

Figure S3. AC-mode AFM images (scan sizes indicated) of the 1:1.5 P3HT: PCBM blend stored in the dark after preparation. Topography (left column) and phase (right column).

Stored in the dark

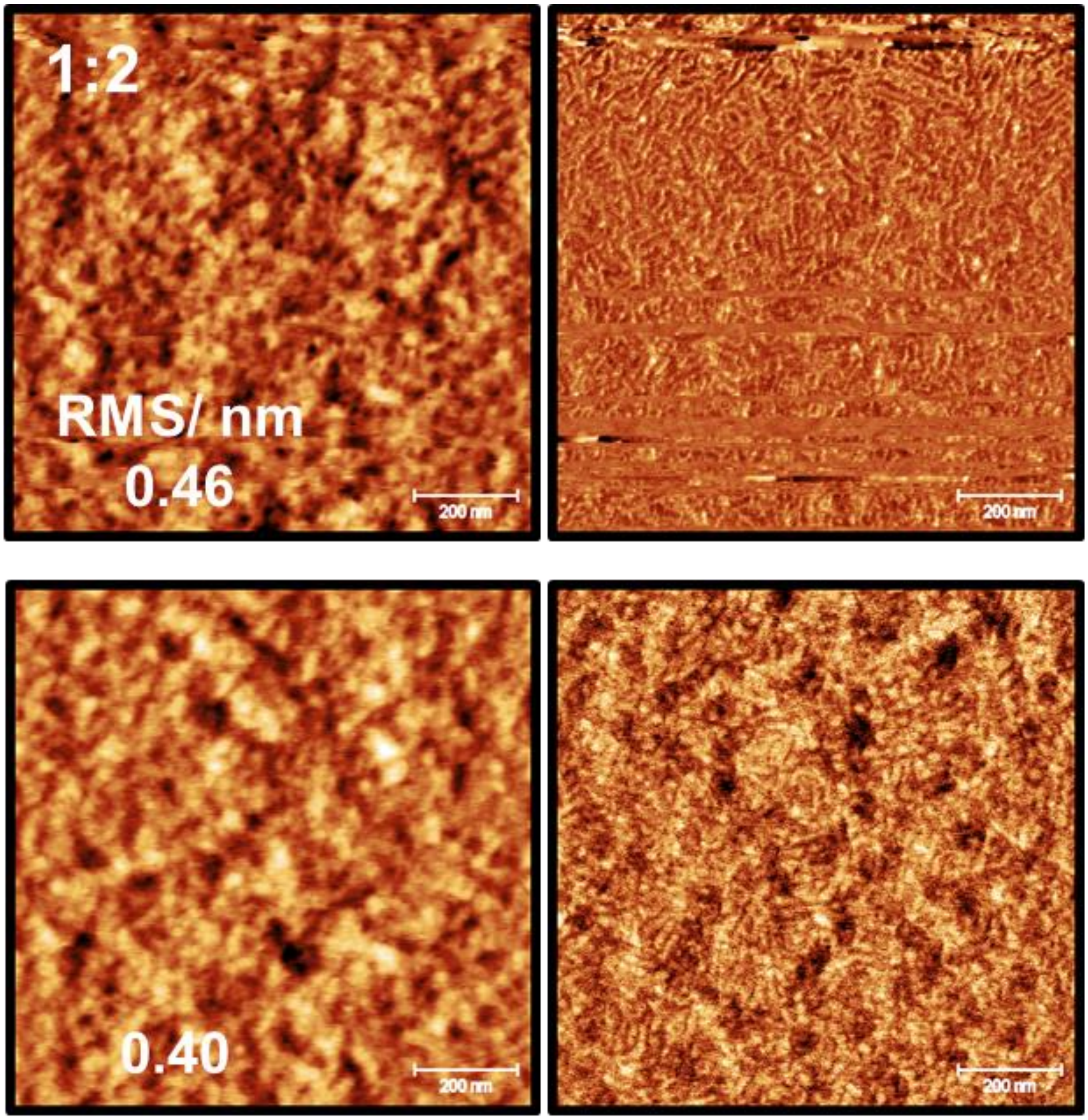

Figure S4. AC-mode AFM images (1 x 1 μm) of the 1:2.0 P3HT: PCBM blend stored in the dark after preparation. Topography (left column) and phase (right column).

Stored in the dark

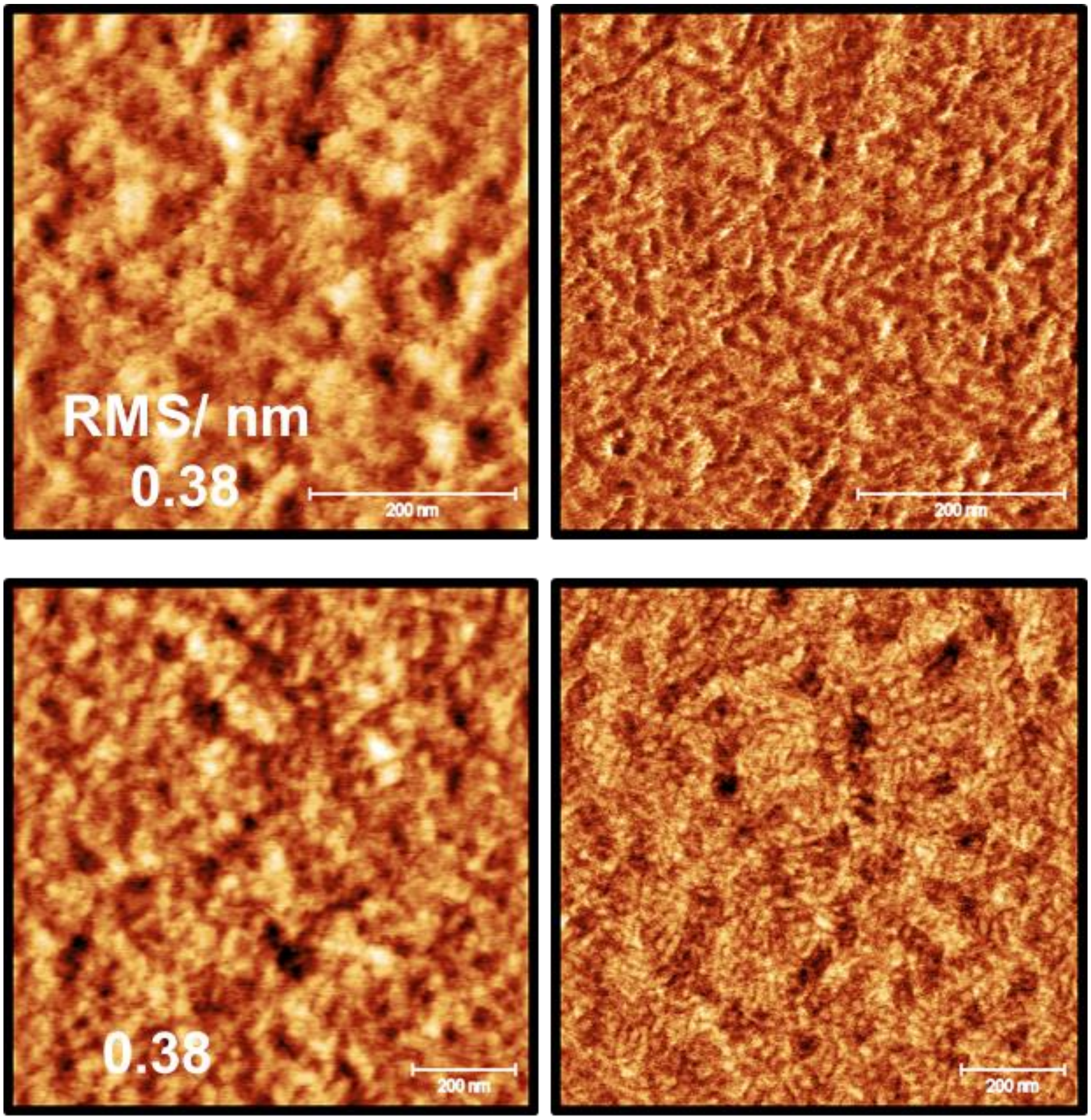

Figure S5. AC-mode AFM images (500 x 500 nm) of the 1:2.0 P3HT: PCBM blend stored in the dark after preparation. Topography (left column) and phase (right column).

Stored under ambient laboratory diffuse light

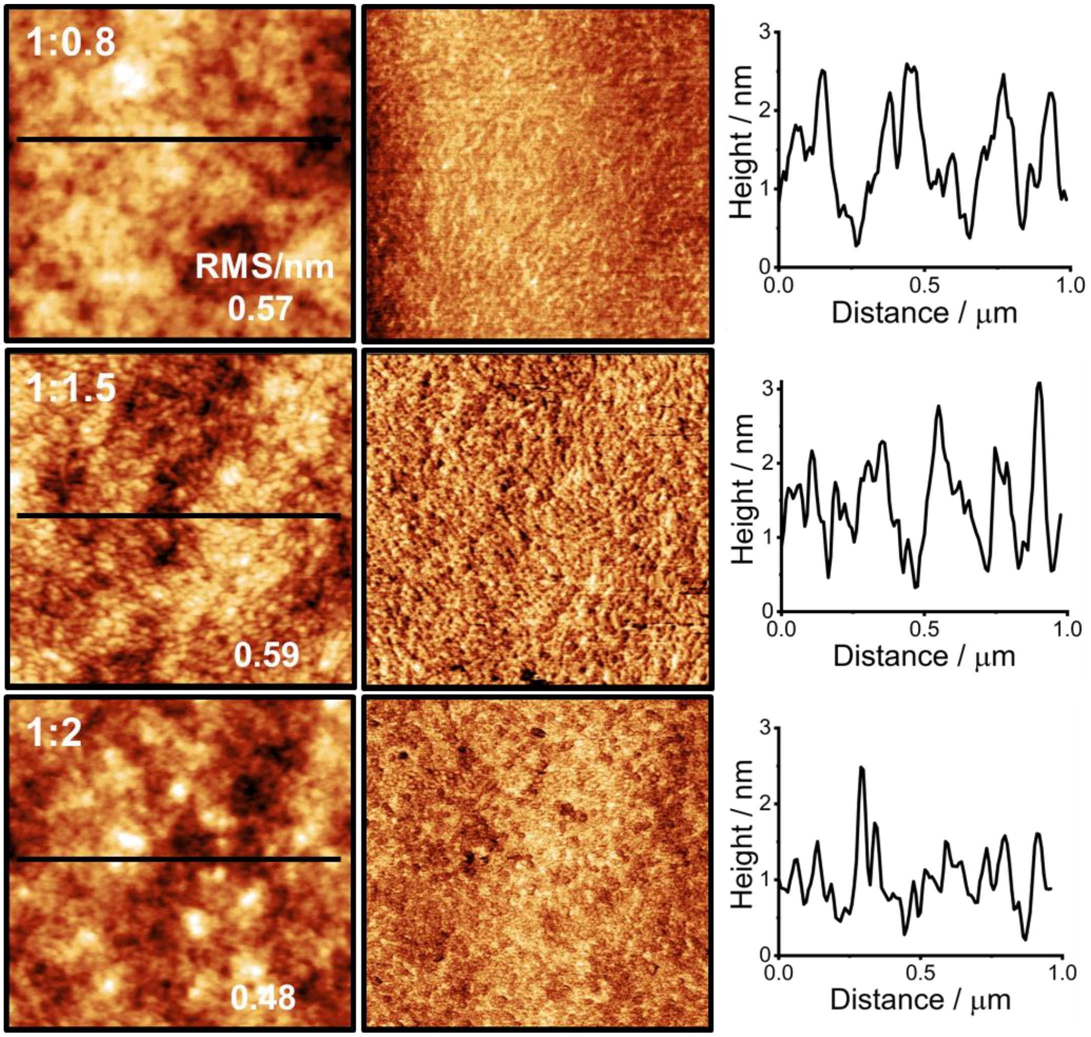

Figure S6. AC-mode AFM images (scan size (1 x 1) µm) of P3HT: PCBM blends left under ambient light conditions and analyzed after 24 hours. Topography (left columns) and phase (right columns) images with corresponding black line profiles extracted from the corresponding topography images. Blends: 1:08 (top row), 1:1.5 (middle row), 1:2 (bottom row). Images acquired using the first eigenmode of the cantilever.

Stored under ambient laboratory diffuse light

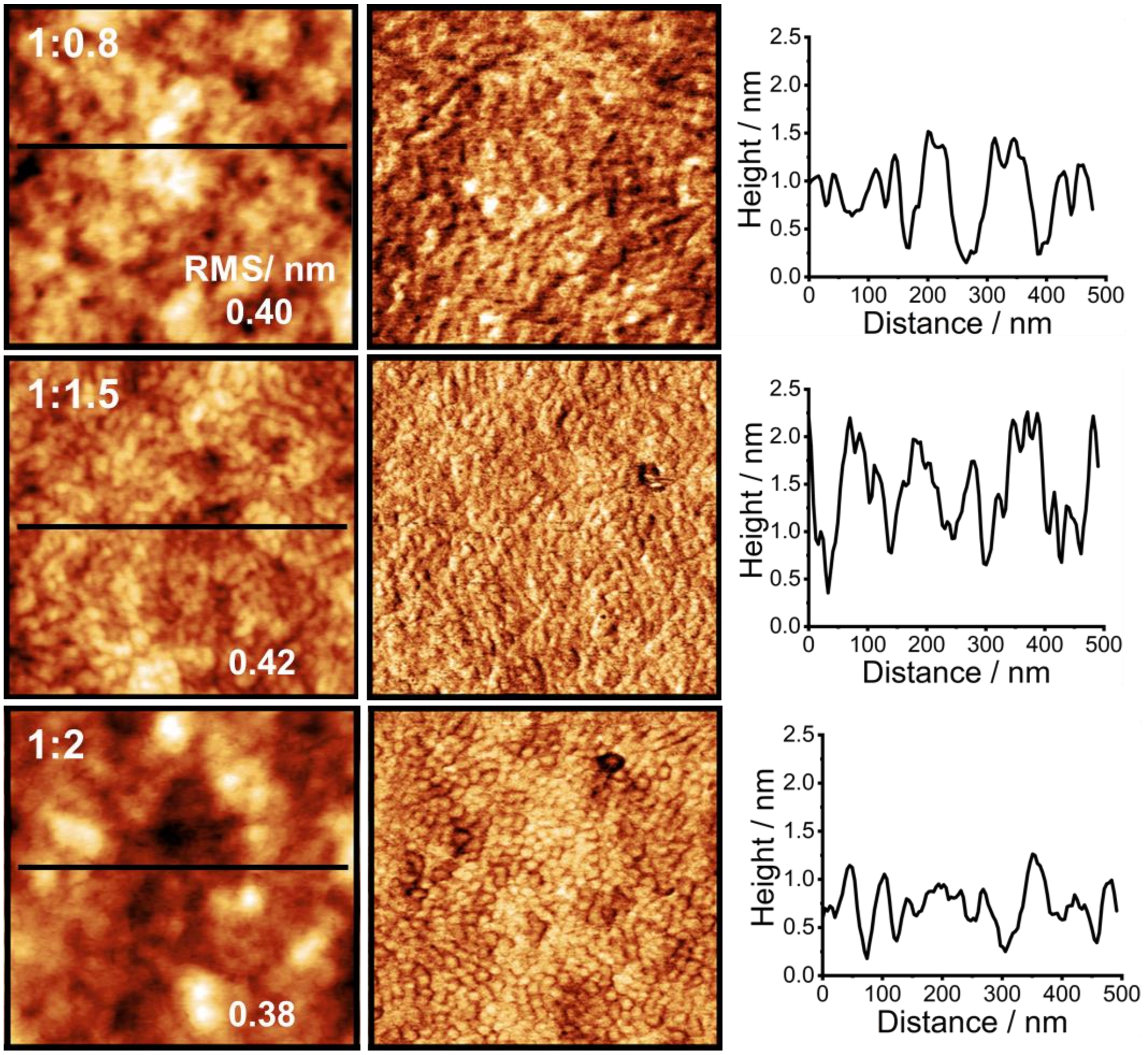

Figure S7. AC-mode AFM images (scan size 500 x 500 nm) of P3HT: PCBM blends left under ambient light conditions and analyzed after 24 hours. Topography (left columns) and phase (right columns) images with corresponding black line profiles extracted from the corresponding topography images. Blends: 1:08 (top row), 1:1.5 (middle row), 1:2 (bottom row). Images acquired using the first eigenmode of the cantilever.

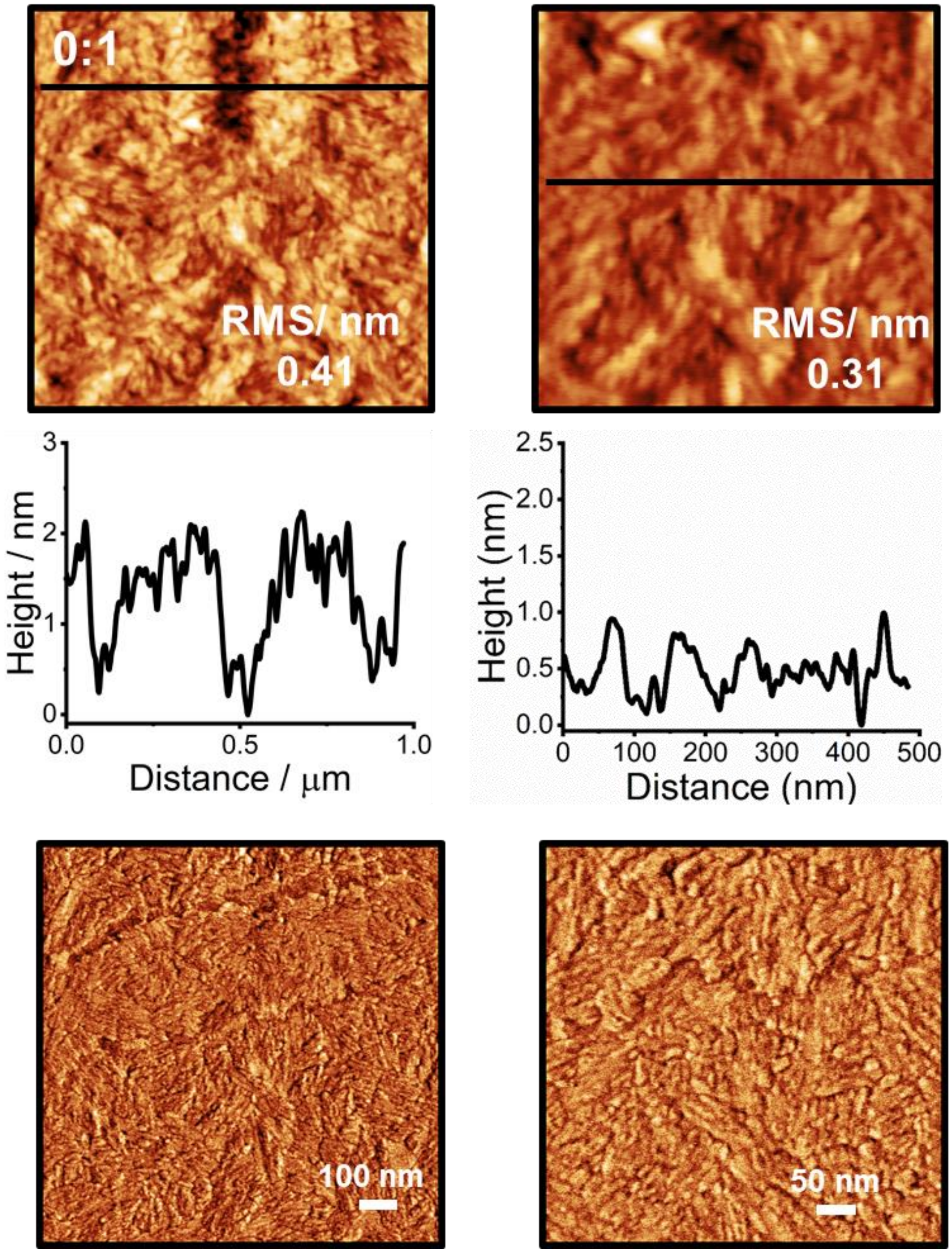

Figure S8. AC-mode AFM images (scan size 1 x 1 μm on the left and 500 x 500 nm on the right) of PCBM. Top row shows topography, the middle row a profile along the line indicated in the topographic map, and bottom row the corresponding phase image.

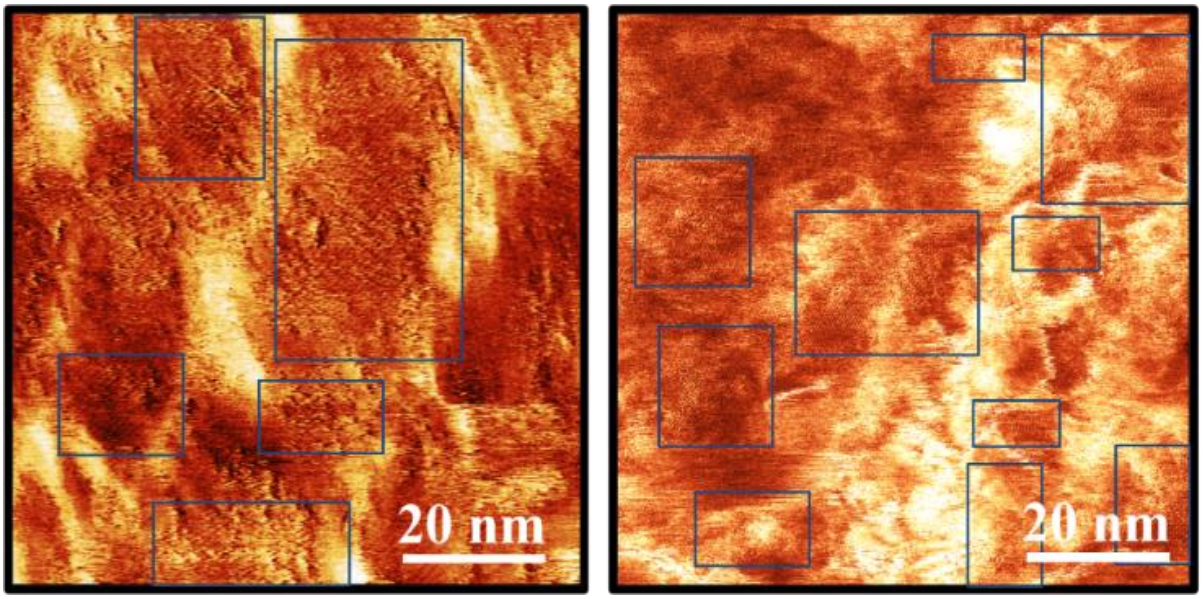

Figure S9. High-resolution AFM phase images of the P3HT film acquired in different zones performed in AC-mode with a cantilever oscillating at the third eigenmode 927 kHz, scan size (100x100) nm. Highlighted regions with blue rectangles show the periodic arrangement of the polymer on the surface of the film.

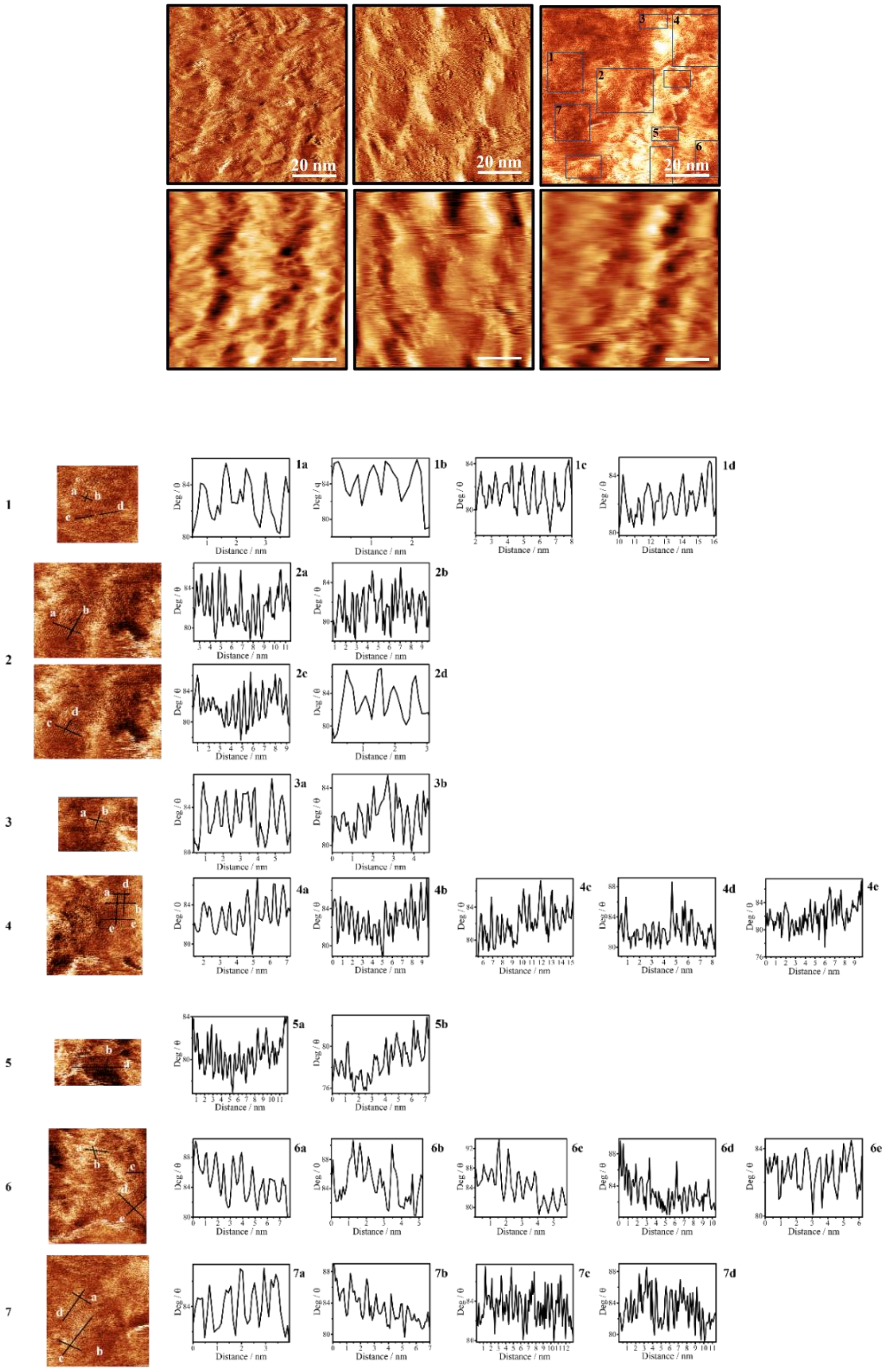

Figure S10. High resolution phase (top row) and topography (bottom row) images of different areas of the film made of the P3HT polymer. Blue rectangles in the phase image on the right show the numbered domains with local order. Such domains are displayed from 1 to 7 with profiles extracted from the corresponding black lines.

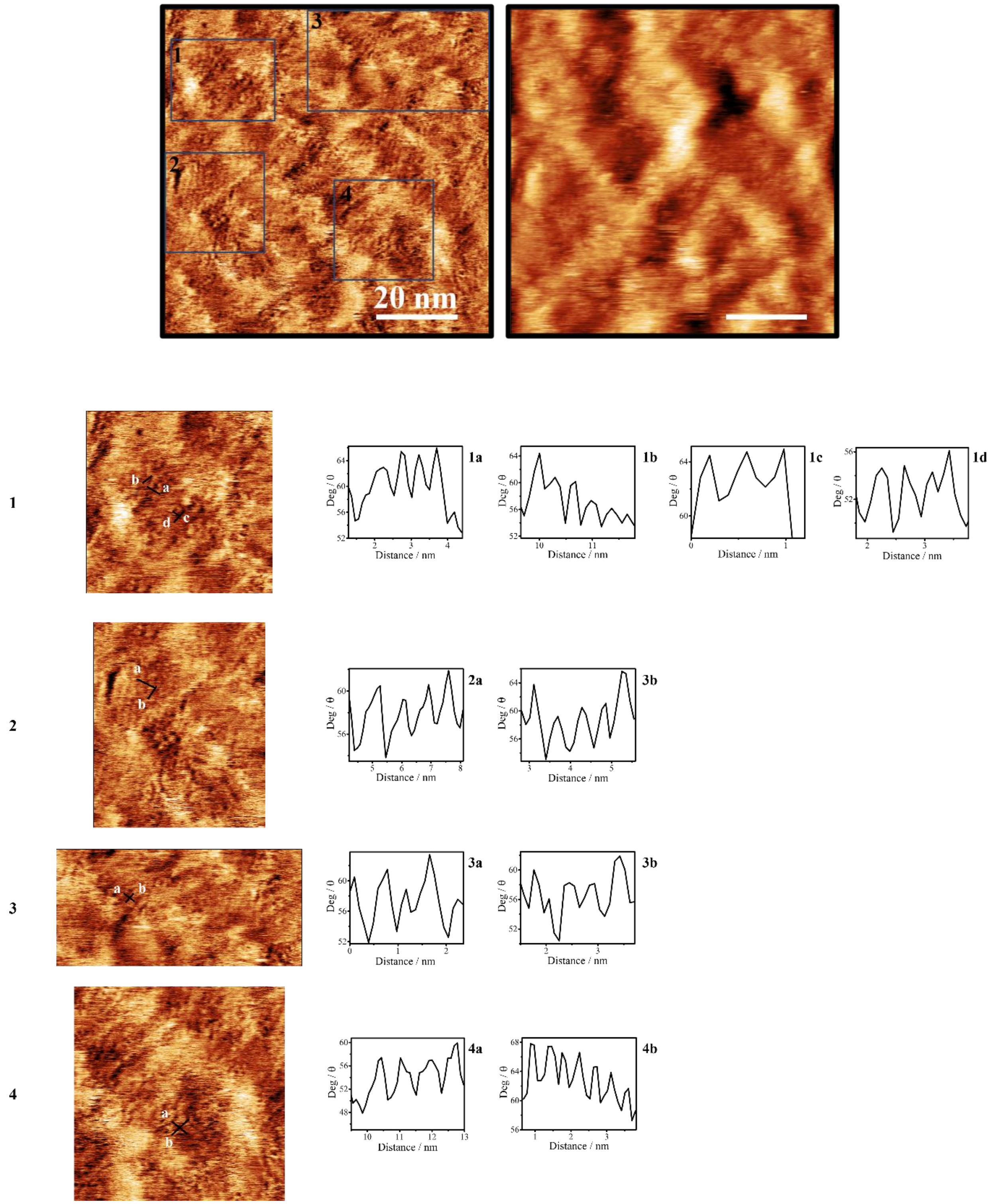

Figure S11. High resolution phase (on the left) and topography (on the right) images of different areas of the film made of the mixture 1:0.8/P3HT:PCBM . Blue rectangles in the phase image show the numbered domains with local order. Such domains are displayed from 1 to 4 with profiles extracted from the corresponding black lines.

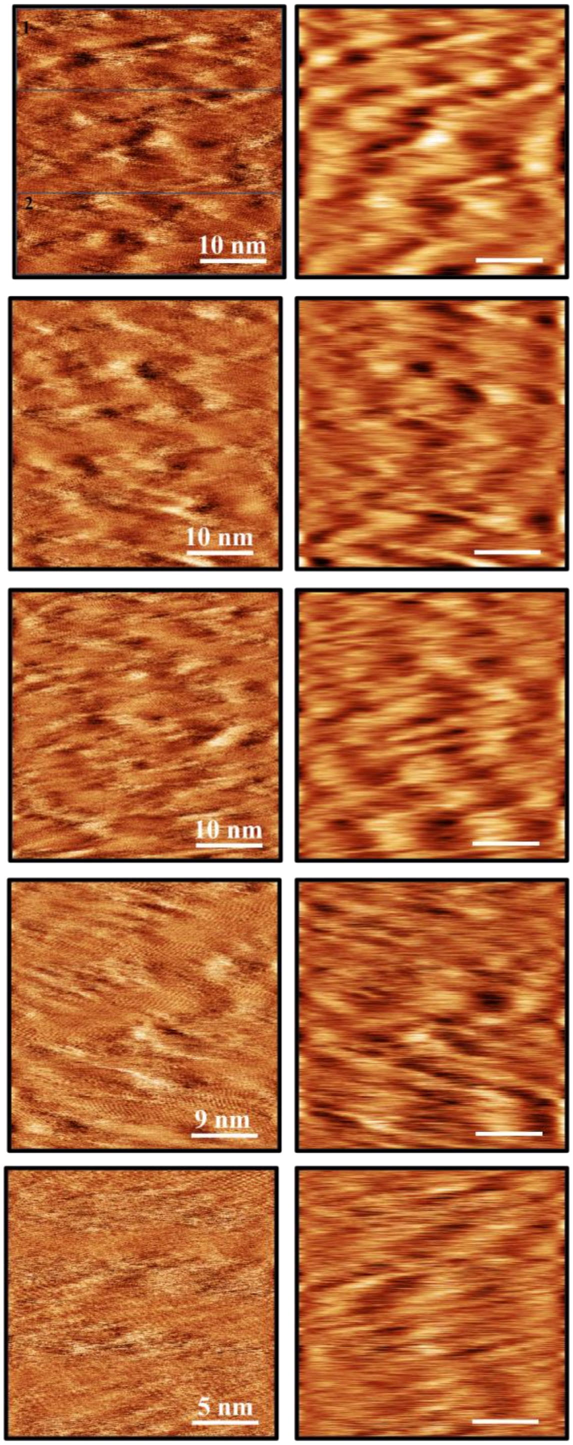

Figure S12. High resolution phase images (on the left) and topography (on the right) of different areas of the film made of the mixture 1:1.5/P3HT:PCBM with different scan sizes. Blue rectangles in the phase image (top row) show the numbered domains with local order. Such domains are numbered 1 and 2 with profiles extracted from the corresponding black lines in Figure S13.

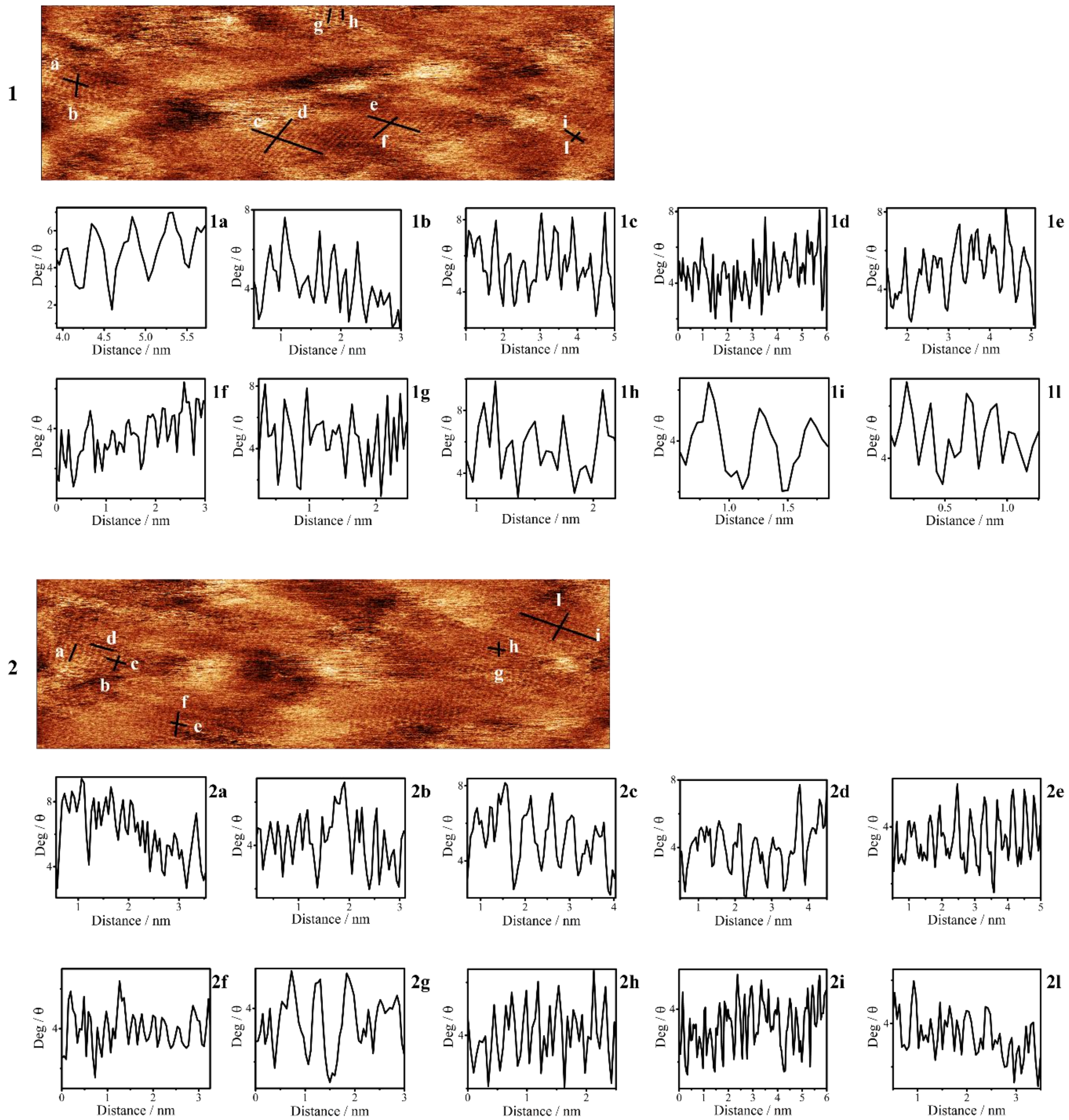

Figure S13. High resolution phase images of different areas of the film made of the mixture 1:1.5/P3HT:PCBM, areas with profiles extracted from the corresponding black lines correspond to blue rectangles numbered 1 and 2 in Figure S12.

X-ray photoelectron spectroscopy (XPS) data

Two Figures S14 and S15 show, respectively, the XPS data of the pristine PCBM and the 1:2/P3HT: PCBM mixture, and Figure S16 shows fitting of the data. A consistent peak-fitting methodology was used to determine the distribution of P3HT and PCBM from their specific atomic elements in the mixture. Figure S14. shows the high resolution XPS spectra of the C 1s and the O 1s regions of the PCBM surface. Peak positions are referenced to the standard binding energy (B.E.) peak position of the C 1s peak at 285.0 eV. The C 1s region of pristine PCBM decomposed into four component peaks; the peak at 285.0 eV (59.2 %) corresponds to the conjugated C atoms of the fullerene and the phenyl group, the dominant C species in molecule; the component at 285.6 eV (22.6 %) is assigned to the saturated C atoms of the butyl chain; the component at 287.1 eV (10.4 %) corresponds to the methoxy C of the methyl ester group (-O-CH3); the component at 289.0 eV (7.9 %) is assigned to carbonyl C from the methyl ester; finally the really broad component at 291.0 eV is associated to the C 1s peak. The O 1s spectrum is decomposed into two component peaks pertaining to the methyl ester group. The component at 533.7 eV (29.6 %) corresponds to methoxy oxygen (C-O-CH_3), and the other at 532.4 eV (70.36 %) represents the carbonyl O (C=O). These peak positions are consistent with findings from previous XPS studies.[1] The relative C 1s and O 1s peak areas in this PCBM surface is 8:1 and the theoretical C: O ratio in bulk PCBM is 36:1. The lower ratio in the XPS spectra shows that PCBM preferentially orients with its methyl ester group closer to the surface and the fullerene part near the PEDOT: PSS sub-layer in order to minimize its surface energy. Figure S15 and Table S1 show the C 1s and O 1s spectra and the S 2p spectrum for the mixture P3HT: PCBM (1:2). The presence of the electron donor P3HT that interact with electron acceptor fullerene derivative can explain the variation of the peaks position for both the C and O in terms of few

eV. Furthermore, the XPS spectrum showing the S 2p region confirms the presence of the P3HT at the top of the active layer into the film mixture. The component peaks at 164.2 and 165.4 eV are associated to the thiophene ring and the component broad peak at 168 eV is assigned to the sulfonate or to eventual contamination from the sub-layer.[1,2]

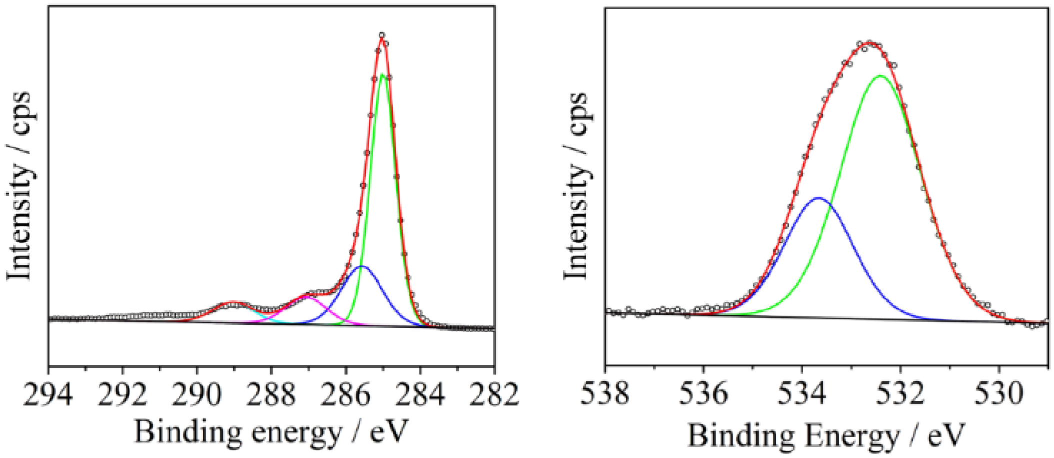

Figure S14. XPS spectra (counts per second, cps vs binding energy, B.E.) for pristine PCBM: C 1s (left) and O 1s (right). Experimental data (spots line), whole curve fit (red line), different component (colored lines).

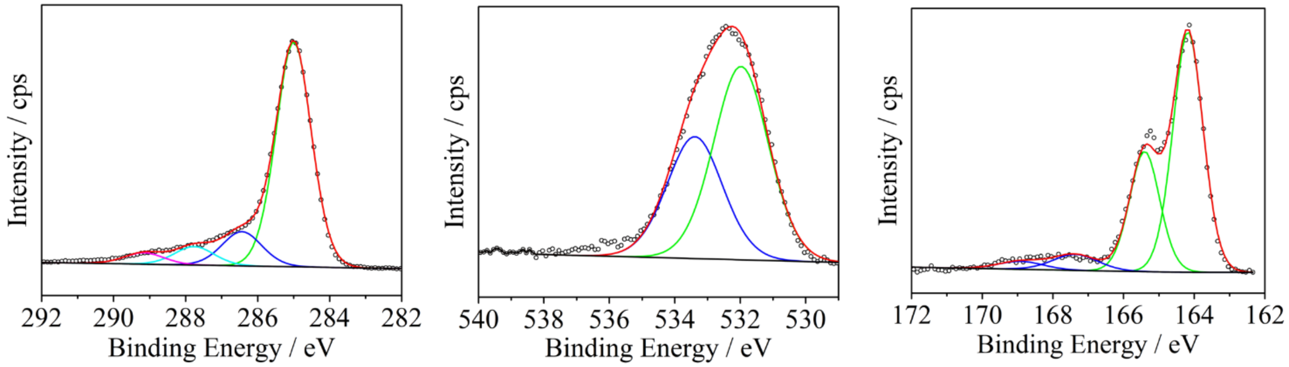

Figure S15. XPS spectra (counts per second, cps vs binding energy, B.E.) for P3HT: PCBM (1:2). From the left: C 1s, O 1s and S 2p. Experimental data (spots line), whole curve fit (red line), different component (colored lines).

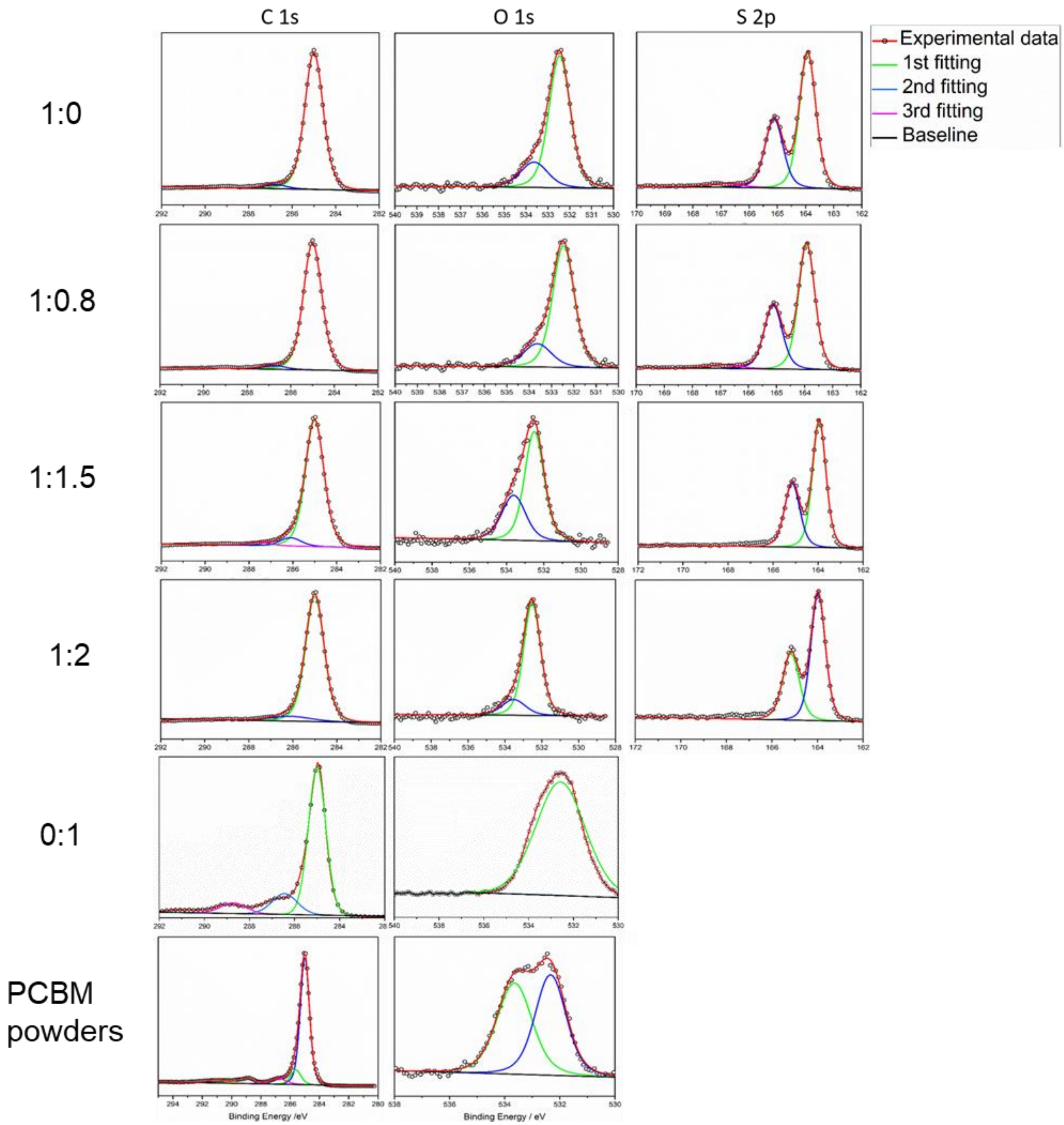

Figure S16. Fitting of the XPS data for all the mixtures and for the pure P3HT film and PCBM powders.

Table S1. Binding energies from high resolution XPS analysis.

		BE_{PCBM} / eV	$BE_{P3HT:PCBM}$/ eV	ΔBE/ eV
**C 1s**	C60, phenyl	285.0	285.0	0.0
	C butyl chain	285.6	286.4	0.9
	Methoxy C	287.1	287.8	0.7
	Carbonyl	289.0	289.2	0.1
	C other	290	-	-
**O 1s**	Carbonyl C=O	532.4	532.0	0.4
	Methoxy C-O-CH_3	533.7	533.4	0.3

Binding energies for C 1s and O 1s for the pristine PCBM and for the sample P3HT: PCBM with ratio 1: 2, and difference in energies (eV) between the spectra for each element.

Table S2. Three individual XPS analyses with calculated peak areas. PCBM values correspond to powders of the material.

D:A	Area C 1s	Area O 1s	Area S 2p	C:O	C:S
1:0	16524.87	1012.56	2669.66	16.31989	6.18988
1:0.8	14865.16	2121.82	1624.85	7.00585	9.68912
1:1.5	19540.63	1643.63	2506.03	11.8887	7.79744
1:2	9613.69	1246.17	1336.37	7.71459	7.19388
0:1	21152.05	6638.11	0	3.18646	--
PCBM	18307.41	2280.54	0	8.02766	--

D:A	Area C 1s	Area O 1s	Area S 2p	C:O	C:S
1:0	15082.42	1731.05	2344.11	8.71287	6.43418
1:0.8	15734.84	2177.83	1655.98	7.22501	9.50183
1:1.5	16393.84	779.42	2402.16	21.03338	6.82462
1:2	9337.43	1198.11	1239.71	7.79347	7.53195
0:1	21131.09	6704.1	0	3.15197	--
PCBM	16981.69	1987.37	0	8.54481	--

Table S2 contd.. Three individual XPS analyses with calculated peak areas. PCBM values correspond to powders of the material.

D:A	Area C 1s	Area O 1s	Area S 2p	C:O	C:S
**1:0**	15641.79	1124.77	2875.67	13.90666	5.43936
**1:0.8**	13702.71	1441.53	1793.74	9.50567	7.63918
**1:1.5**	13954.51	520.37	2126.35	26.81652	6.56266
**1:2**	9847.42	1276.92	1326.9	7.71185	7.42137
**PCBM**	16869.15	1914.73	0	8.8102	--

Table S3. Three individual XPS analyses with calculated peak areas. PCBM values correspond to powders of the material.

D:A	Average C:O	Average C:S	C:O ratio	C:S ratio
**1:0**	12.97980	6.02114	13:1	6:1
**1:0.8**	7.91217	8.94337	8:1	9:1
**1:1.5**	19.91286	7.06157	20:1	7:1
**1:2**	7.73997	7.3824	8:1	7:1
**0:1**	3.16921	--	3:1	--
**PCBM**	8.46089	--	8:1	--

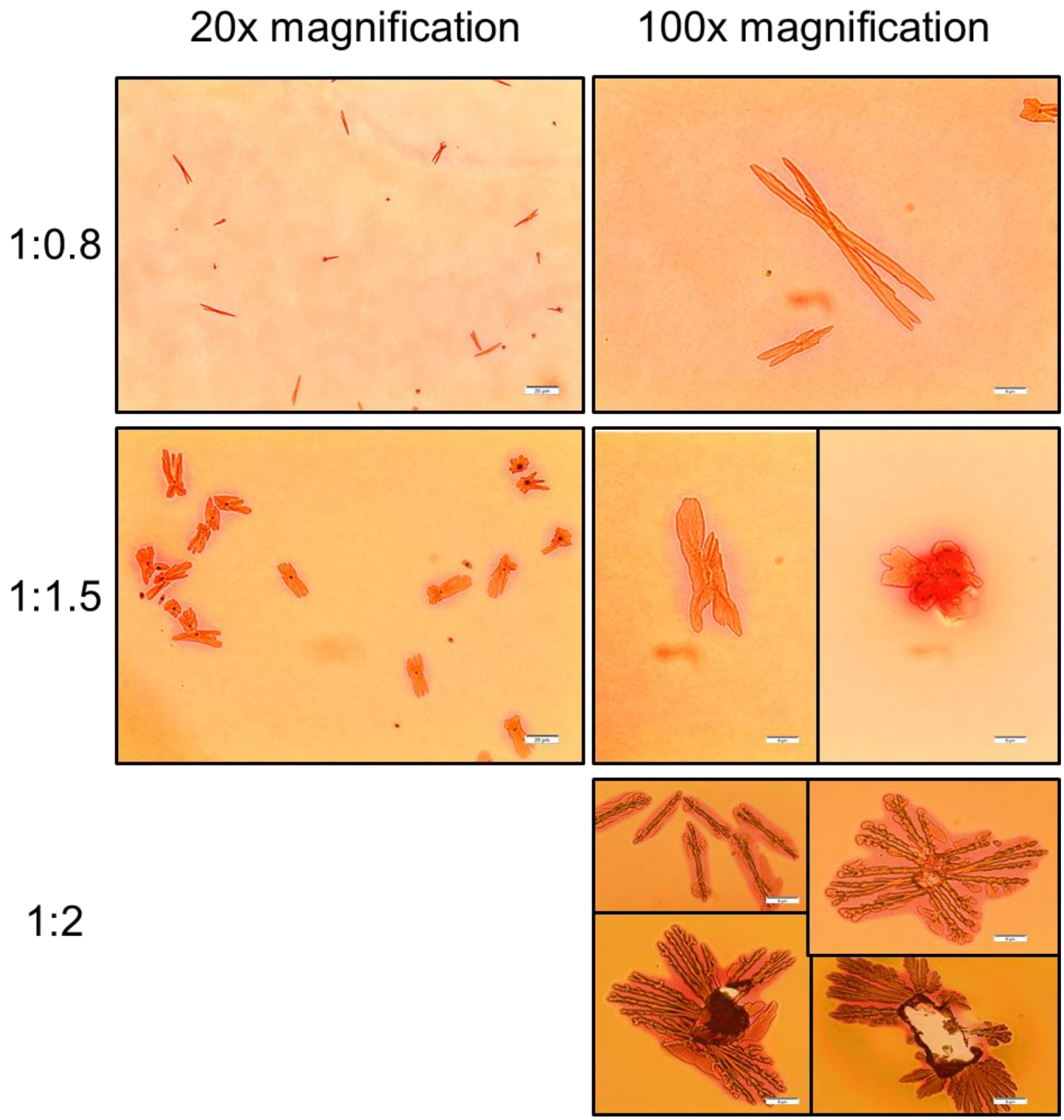

Figure S17. Optical micrographs of the blends 1:0.8 (top row), 1:1.5 (middle row) and 1:2 (bottom row) /P3HT:PCBM at different magnifications.

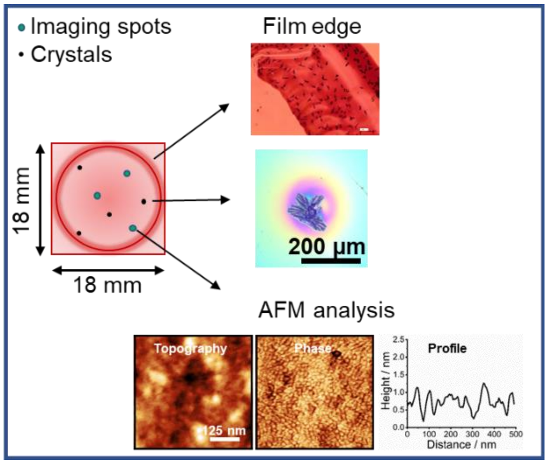

Figure S18. Drawing of a typical sample showing how the analysis was performed. Edges were avoided due to inhomogeneity of the spin coating procedure. Images were taken in the middle of the sample and far from crystals.

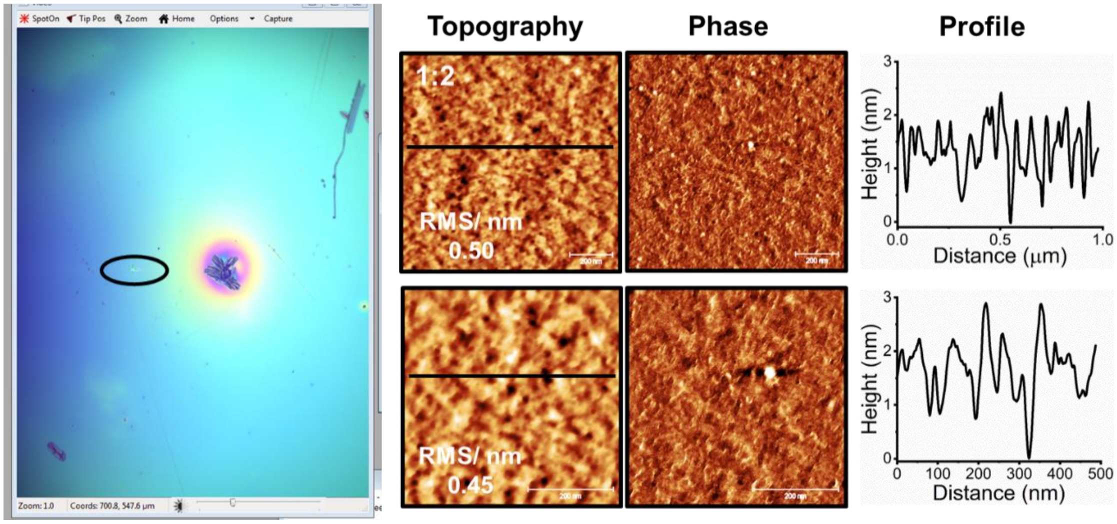

Figure S19. Optical micrograph of the surface investigated showing a PCBM crystal for the sample 1:2/P3HT:PCBM and highlighted area with a black circle where the scan was performed (on the left). AFM topography and phase images of the spot investigated at (1x1) µm (top right) and (500x500) nm (bottom right) with corresponding line profiles taken from the topography images.

REFERENCES

(1) Busby, Y.; List-Kratochvil, E. J. W.; Pireaux, J. J. Chemical Analysis of the Interface in Bulk-Heterojunction Solar Cells by X-Ray Photoelectron Spectroscopy Depth Profiling. *ACS Applied Materials and Interfaces* **2017**, *9* (4), 3842–3848. https://doi.org/10.1021/acsami.6b14758.

(2)釐Paternò, G. M.; Robbiano, V.; Fraser, K. J.; Frost, C.; García Sakai, V.; Cacialli, F. Neutron Radiation Tolerance of Two Benchmark Thiophene-Based Conjugated Polymers: The Importance of Crystallinity for Organic Avionics. *Scientific Reports* **2017**, *7*, 41013. https://doi.org/10.1038/srep41013.
